# Exploiting the Degradation Mechanism of NCM523∥ Graphite Lithium‐Ion Full Cells Operated at High Voltage

**DOI:** 10.1002/cssc.202002113

**Published:** 2020-11-10

**Authors:** Sven Klein, Peer Bärmann, Thomas Beuse, Kristina Borzutzki, Joop Enno Frerichs, Johannes Kasnatscheew, Martin Winter, Tobias Placke

**Affiliations:** ^1^ University of Münster, MEET Battery Research Center Institute of Physical Chemistry Corrensstr. 46 48149 Münster Germany; ^2^ Helmholtz Institute Münster, IEK-12 Forschungszentrum Jülich GmbH Corrensstr. 46 48149 Münster Germany; ^3^ University of Münster, Institute of Physical Chemistry Corrensstr. 30 48149 Münster Germany

**Keywords:** lithium-ion batteries, degradation mechanisms, electrode materials, metal deposition, single-crystals

## Abstract

Layered oxides, particularly including Li[Ni_*x*_Co_*y*_Mn_*z*_]O_2_ (NCM*xyz*) materials, such as NCM523, are the most promising cathode materials for high‐energy lithium‐ion batteries (LIBs). One major strategy to increase the energy density of LIBs is to expand the cell voltage (>4.3 V). However, high‐voltage NCM∥ graphite full cells typically suffer from drastic capacity fading, often referred to as “rollover” failure. In this study, the underlying degradation mechanisms responsible for failure of NCM523∥ graphite full cells operated at 4.5 V are unraveled by a comprehensive study including the variation of different electrode and cell parameters. It is found that the “rollover” failure after around 50 cycles can be attributed to severe solid electrolyte interphase growth, owing to formation of thick deposits at the graphite anode surface through deposition of transition metals migrating from the cathode to the anode. These deposits induce the formation of Li metal dendrites, which, in the worst cases, result in a “rollover” failure owing to the generation of (micro‐) short circuits. Finally, approaches to overcome this dramatic failure mechanism are presented, for example, by use of single‐crystal NCM523 materials, showing no “rollover” failure even after 200 cycles. The suppression of cross‐talk phenomena in high‐voltage LIB cells is of utmost importance for achieving high cycling stability.

## Graphical abstract

**A worst‐case scenario**: Lithium‐ion battery cells operated at high voltage typically suffer from drastic capacity fading. The degradation mechanisms responsible for cell failure are unraveled for NCM523 graphite cells operated at 4.5 V, and degradation is attributed to severe solid electrolyte interphase growth at graphite through deposited transition metals. These deposits induce the formation of Li metal dendrites, which, in the worst case, lead to (micro‐) short circuits.

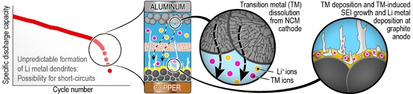

## 1 Introduction

Lithium‐ion batteries (LIBs) are the state‐of‐the‐art battery technology for various application purposes, particularly including electric vehicles (EVs), due to their outstanding characteristics such as the high energy and power density, high energy efficiency, long cycle life, low self‐discharge and high safety compared to other battery technologies.[^1^, ^2^, ^3^, ^4^, ^5^] Despite their advantageous characteristics, LIBs can fulfill only some of the highly demanding requirements in terms of energy density, needed to enable a broad market penetration of EVs, which require driving ranges of at least 500 km.[^2^, ^6^, ^7^] To realize further improvements, intensive research and development efforts for LIBs are mandatory. In particular, the development and inclusion of advanced active materials is believed to be the most suitable option to achieve the aforementioned energy density targets.[^8^, ^9^]

The positive electrode (cathode) has proven to be the bottleneck regarding the energy density of LIBs and significantly contributes to the LIB cell costs, demanding further significant optimization.[^6^, ^10^, ^11^, ^12^] The state‐of‐the‐art cathode materials for high‐energy LIB cells are the layered lithium nickel cobalt manganese oxides Li[Ni_*x*_Co_*y*_Mn_*z*_]O_2_ (*x*+*y*+*z*=1; further abbreviated as: NCM*xyz*), because of their high theoretical capacities of up to around 280 mAh g^−1^.[^7^, ^13^, ^14^, ^15^] A classic example is NCM111, with a practical initial capacity of around 160 mAh g^−1^, at moderate C‐rates.^[14]^ Two major approaches are pursued to increase the amount of extracted lithium from NCM‐based cathode materials to enhance the LIB's energy density. Whereas the first strategy focuses on the increase of the layered oxides’ nickel (Ni) content (e. g., ≥80 %), the second approach addresses the increase of the upper charging cutoff potential (e. g., >4.3 V vs. Li|Li^+^) of low Ni (≤60 %) NCM cathodes to achieve a higher de‐lithiated state and, thus, a higher specific capacity.[^6^, ^14^, ^16^, ^17^, ^18^, ^19^] Based on the thermal stability of NCM523 and NCM811 materials, NCM523 is the more promising candidate for high‐voltage application,^[20]^ delivering a high practical capacity of around 200 mAh g^‐1^ at 0.1 C (cell voltage ≥4.4 V). In general, it is well‐known that commercial NCM‐based LIB cells can deliver a cycle life of several hundreds or even thousands of cycles when operated at a cell voltage ≤4.3 V.[^2^, ^6^] However, the high‐voltage operation of NCM‐based LIB cells is a severe challenge and results in fast and significant capacity fading (e. g., in less than 200 cycles using non‐optimized materials/components), which is sometimes also referred to as “rollover” failure,^[21]^ and has been observed by various researchers.[^22^, ^23^, ^24^, ^25^, ^26^, ^27^] Even though there are approaches to improve the cycling stability (e. g., by cathode electrolyte interphase (CEI) forming electrolyte additives, cathode surface coatings, etc.),[^21^, ^23^, ^24^, ^28^, ^29^] the origin of this failure mechanism and the impact of different cell parameters is not clearly understood so far.

The rapid capacity fading of NCM cathodes can be attributed to various aging effects such as an instable NCM surface, phase transformation, oxidative decomposition of carbonate‐based electrolytes and impedance growth at the cathode surface due to CEI growth.[^18^, ^28^] Another important factor resulting in capacity loss, impedance growth and cell failure of high‐voltage NCM∥ graphite full cells can be associated to transition metal (TM) dissolution from the cathode material (e. g., Co^2+^, Ni^2+^, Mn^2+^), which migrate through the electrolyte/separator and can deposit at the negative electrode (anode) surface.[^15^, ^25^, ^26^, ^27^, ^30^, ^31^, ^32^, ^33^, ^34^, ^35^, ^36^, ^37^] In this context, Joshi et al. studied the impact of small amounts of Ni^2+^, Mn^2+^ and Co^2+^ on the performance of NCM111∥ graphite full cells by adding Ni(TFSI)_2_, Mn(TFSI)_2_ and Co(TFSI)_2_ salts into the LiPF_6_‐based carbonate electrolytes.^[31]^ However, their model experiment can hardly be compared to practical LIB cells, as large amounts of dissolved TM ions were present right after cell assembly and thus influenced the solid electrolyte interphase (SEI)^[38]^ formation and growth right after cell assembly. Thereby, they observed the formation of a thick, rough SEI layer on graphite particles, whereas the particle surface was smooth without adding these metal ion salts.^[31]^ In regard of Mn^II^ deposition at the anode surface, Zhan et al. demonstrated that the choice of anode material can significantly influence the capacity retention and cycle life.^[39]^ In case of Li metal^[40]^ or graphite anodes,^[41]^ operating at low potentials vs. Li|Li^+^, the deposited Mn^II^ showed a strong impact on performance, whereas Li_4_Ti_5_O_12_, having a high operation potential, only showed a negligible effect on performance by Mn^II^ deposition.^[39]^ Additionally, Gilbert et al. showed that the majority of capacity loss in high‐voltage LIB cells can be contributed to irreversible lithium loss in the SEI at the anode, induced by TM deposition.^[26]^

**Figure 1:**
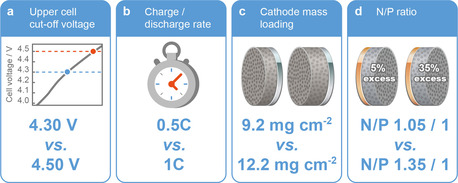
Investigation of different electrode and cell parameters in high‐voltage NCM523∥ graphite full cells in regard of their impact on cell performance and transition metal deposition at the graphite anode: Impacts of (a) upper cutoff voltage (4.30 V vs. 4.50 V), (b) charge–discharge rate (at 4.50 V), (c) cathode mass loading (at 4.50 V), and (d) N/P capacity balancing ratio (at 4.50 V).

The impact of TM dissolution/deposition on the performance of high‐voltage LIB cells is not completely understood, especially with respect to various cell characteristics influencing TM dissolution/deposition and inducing further parasitic side reactions. In this work, we carefully examine different practical electrode and cell parameters in regard of TM deposition and their impact on the cycle life in high‐voltage NCM523∥ graphite LIB full cells. The focus of this work mainly lies on the systematic investigation of the graphite anode surface and of the graphite particles by means of SEM and EDX. The studied electrode and cell parameters are summarized in Figure 1.

## Experimental Section

### Electrode preparation

The NCM523‐based cathodes and the graphite‐based anodes were prepared in large‐scale at an in‐house battery line. The cathodes consisted of 95 wt % NCM523 (polycrystalline NCM523; Custom Cells Itzehoe GmbH), 3 wt % polyvinylidene fluoride (PVdF) binder (Solef 5130, Solvay) and 2 wt % carbon black (Super C65, Imerys Graphite & Carbon) and were cast onto aluminum foil (15 μm; Nippon Foil). The used solvent was N‐methyl‐2‐pyrrolidone (NMP, Sigma Aldrich, purity: 99.5 %). Two different mass loadings were prepared: a) 9.2 mg cm^−2^ and b) 12.2 mg cm^−2^. In addition, a single‐crystal NCM523 material (commercial supplier) was analyzed at the same conditions (see also Supporting Information).

The anodes consisted of 95 wt % graphite (SG3, natural graphite, SGL Carbon), 1.5 wt % styrene‐butadiene‐rubber (SBR; SB5521, LIPATON, Polymer Latex GmbH), 3 wt.% Na‐CMC (Walocel CRT 2000 PPA12; Dow Wolff Cellulosics) and 0.5 wt % carbon black (Super C65, Imerys Graphite & Carbon) and were cast onto copper foil (10 μm; Nippon Foil). The used solvent was deionized water. Two different mass loadings were prepared for the anodes: a) 8.8 mg cm^−2^ and b) 6.9 mg cm^−2^. After drying and calendaring of the electrode sheets, they were punched into circular Ø14 mm (cathode) and Ø15 mm (anode) discs. The electrodes were dried in a vacuum oven at 100 °C under reduced pressure. Additionally, Ø12 mm electrodes for both the anode and cathode were punched out of the electrode sheets and dried at the same conditions for investigation of the individual anode and cathode potentials in three‐electrode T‐cells. The electrode balancing, N/P ratio, was set to approximately 1.35 / 1.00 or 1.05 / 1.00, depending on the experiment, as stated herein. All electrode parameters are summarized in Table S1 (Supporting Information).

### Cell assembly

2032‐type coin cells (two‐electrode configuration^[42]^) were assembled to investigate transition metal (TM) dissolution from the NCM523 cathode and the TM deposition at the graphite anode in NCM523∥ graphite full cells. The Ø15 mm anode disc was separated by a Celgard 2500 separator (polypropylene, one layer) from the Ø14 mm cathode disc, which was soaked with 40 μL LP57 electrolyte (1 M LiPF_6_ in EC/EMC 3 : 7 by weight, BASF SE; purity: battery grade).

Swagelok‐type T‐Cells (three‐electrode configuration^[42]^) were assembled to investigate the individual anode and cathode potentials for the high‐voltage NCM523∥ graphite full cells,^[42]^ using Li metal (Ø8 mm) as reference electrode (RE; Albemarle Corporation; purity: battery grade). Therefore, anode and cathode discs with the same size (both Ø12 mm) and a Celgard 2500 separator (one layer, thickness: 25 μm, Ø13 mm for anode/cathode; Ø10 mm for the RE) were used. The electrolyte amount in these three‐electrode cells was 40 μL and 30 μL for the Ø13 mm and Ø10 mm Celgard separators, respectively.

For comparison, different separator materials were also compared in NCM523∥ graphite full cells, that is, a polyolefin separator [Freudenberg, FS2190, 3 layers, thickness of one layer≈230 μm (not compressed); electrolyte: 200 μL] and a glass fiber separator [Whatman, grade GF/D, 1 layer; thickness≈680 μm (not compressed); electrolyte: 200 μL]. In addition, NCM523||Cu cells were evaluated using different electrolytes: (I) LP57+0.1 mM LiTFSI (3M, purity: 99.95 %), (II) LP57+0.05 mM Ni(TFSI)_2_ (Alfa Aesar), (III) LP57+0.05 mM Co(TFSI)_2_ (Alfa Aesar), and (IV) LP57+0.05 mM Mn(TFSI)_2_ (Alfa Aesar). For the NCM523||Cu cells, a 12 mm NCM523 cathode and a 15 mm Cu foil (thickness: 10 μm; Nippon Foil) was used, both were separated by a Celgard 2500. The electrolyte amount of the different electrolytes was 50 μL. Besides the standard electrolyte (LP57), the impact of electrolyte additives is demonstrated by using (I) LP57+2 wt.% vinylene carbonate (VC; BASF SE; purity: battery grade) and (II) LP57+1 wt % lithium difluorophosphate (LiDFP; Shenzhen CapChem Tech. Co. Ltd.).

### Constant current‐constant voltage charge–discharge cycling

The electrochemical charge‐discharge cycling performance of NCM523∥ graphite full cells was studied via constant current (CC) charge‐discharge cycling on a Maccor 4000 battery testing system in cell voltage ranges between a) 2.8 V–4.3 V, b) 2.8 V–4.45 V and c) 2.8 V–4.5 V. The cell formation conditions consisted of one cycle at 0.1 C and one cycle at 0.2 C. Afterwards, the cells were cycled with 1 C (1 C=170 mA g^−1^ at 4.3 V; 1 C=185 mA g^−1^ at 4.45 V; 1 C=190 mA g^−1^ at 4.5 V) or 0.5 C (95 mA g^−1^ at 4.5 V) depending on the respective experiment. After each charge step, a constant voltage (CV) step was performed with the limiting conditions of either achieving a time limit of maximal 30 min or when the specific current reaches values below 0.05 C. All electrochemical studies were performed in climatic chambers at 20 °C. At least three cells were evaluated for each study to ensure a high reproducibility, which is indicated by error bars in the respective Figures. Further details are summarized in Table S2 (Supporting Information). To investigate the Li metal deposition in NCM523∥ Cu cells, the cells were charged to 4.2 V (at 0.1 C or 1 C) and disconnected to examine the Li metal deposits via SEM and EDX.

### SEM and EDX investigations

The investigation of the surface morphology of the cycled graphite anodes (after 100 cycles) was performed by a Zeiss Auriga electron microscope and EDX was carried out with an accelerating voltage of 20 kV with an energy‐dispersive X‐ray detector (X‐MaxN 80 mm^2^, Oxford Instruments). Prior to analysis, the cells were disassembled in dry atmosphere (dry room) and the anode surfaces were rinsed with 1 mL EMC. After a short drying period under reduced pressure, the electrodes were transferred into the SEM advice via a vacuum sealed sample holder to avoid any contact to moisture.

### ^7^Li MAS NMR measurements

For the ^7^Li MAS NMR measurements the de‐lithiated graphite electrode (after 100 cycles) was scratched from the copper current collector using a ceramic scalpel after rinsing with 1 mL of EMC. Afterwards, the extracted electrode was ground and diluted 1 : 4 by weight with MgO to prevent EDDY currents and additional heating of the sample during the NMR measurement. The ^7^Li MAS NMR spectra were recorded on 200 MHz Bruker DSX spectrometer equipped with a 4.70 T wide bore magnet using a 2.5 mm Bruker MAS probe. The ^7^Li shifts were referenced to 1 mol L^−1^ lithium chloride solution and its isotropic chemical shift was set δ_iso_=0 ppm. For the investigation of the Li‐species one‐pulse experiments were performed and the recycle delay was set to 50 ms. Processing and analysis of the NMR data were done using the Topspin and DmFit^1^ software packages.^[43]^

## 2 Results and Discussion

### 2.1 Impact of the upper cutoff voltage on the cycling performance of NCM523∥ graphite full cells

**Figure 2:**
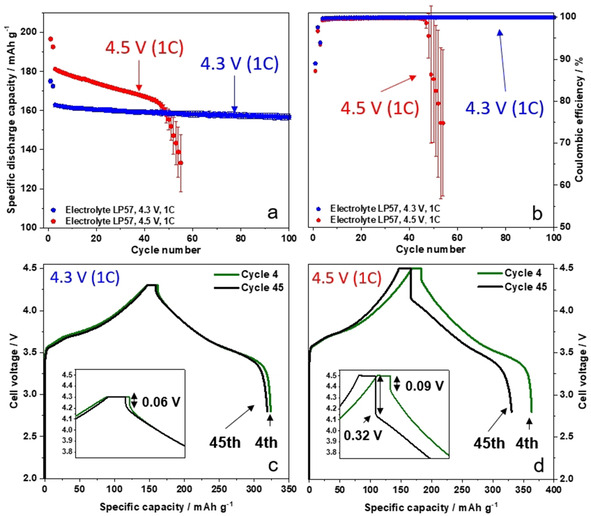
(a,b) Comparison of the charge‐discharge cycling performance of NCM523∥ graphite full cells (coin cells, two‐electrode configuration) in cell voltage ranges of 2.8–4.3 V and 2.8–4.5 V, showing (a) the discharge capacities and (b) the Coulombic efficiencies [cathode mass loading: 12.2 mg cm^−2^; charge–discharge cycling rate: 1 C (=170 mA g^−1^ at 4.3 V and=190 mA g^−1^ at 4.5 V); N/P ratio=1.35 / 1.00 at 4.5 V; 1.15 / 1.00 at 4.3 V and 1.50 / 1.00 at 4.3 V (see also Figure S3)]; (c,d) Corresponding charge–discharge cell voltage profiles of NCM523∥ graphite full cells in cell voltage ranges of (c) 2.8–4.3 V and (d) 2.8–4.5 V, showing selected cycles (4^th^ and 45^th^ cycle).

First, the impact of the upper cutoff voltage (4.30 V vs. 4.50 V) on the electrochemical performance of NCM523∥ graphite full cells and on the “aging” and possible changes of the graphite anode surface was systematically evaluated. Photographs of positive and negative electrodes and separators extracted from NCM523∥ graphite full cells cycled for 100 cycles at cutoff voltages of 4.3 V and at 4.5 V are presented in Figures S1a,b and S2 (Supporting Information). The electrochemical charge‐discharge cycling performance is shown in Figure 2a and b in terms of discharge capacity and Coulombic efficiency (*C*_Eff_), respectively. The first practical capacities at 4.3 V and at 4.5 V are approximately 163 mAh g^−1^ (1 C, 170 mA g^−1^) and 183 mAh g^−1^ (1 C, 190 mA g^−1^), respectively. The combination of high‐voltage operation and –induced by the high voltage – high practical capacity after the formation cycles fulfills the requirements of a high‐energy LIB cell. Besides the high discharge capacity at 4.5 V, the cycling performance already suffers from a severe capacity fading and a dramatic capacity drop, often referred to as “rollover” failure,^[21]^ after roughly 50 cycles. In contrast, the performance at 4.3 V is relatively stable with a capacity retention of around 97 % after 100 cycles. The performance of cells operated at 4.3 V was evaluated in a broad N/P ratio range from 1.15 / 1.00 to 1.50 / 1.00. The two extremes are shown in Figure 2a and Figure S3a. Nevertheless, the cycling stability of the 4.3 V cells remains nearly unaffected (Figure S3a), which is in strong contrast to cells operated at 4.5 V.

The rapid cell failure for LIB cells operated at high‐voltage has been reported by many other researchers,[^26^, ^27^] however, has not been understood so far. We postulate here, that the fast capacity drop at high‐voltage operation (Figure 2a) in combination with the strong drop in the *C*_Eff_ (Figure 2b) can be a hint for the formation of Li metal dendrites [a specific morphology of high surface are lithium (HSAL)][^44^, ^45^] at the graphite negative electrode, causing – in the worst case – a short circuit due to the dendrite penetration through the separator.^[46]^ To investigate the high‐voltage rollover failure in view of Li metal dendrite formation, the cells at 4.3 V (reference cell) and at 4.5 V were disassembled and the individual components were carefully extracted. Figure S1 shows the photographs of the extracted electrodes. The cathode surface shows no visible deposits/decomposition layers for both cases (4.3 V and 4.5 V). In comparison, the anode surfaces cycled at 4.5 V exhibit significantly visible thick deposits (Figure S1b) compared to the anode in cells charged to 4.3 V (Figure S1a). Additionally, small black deposits stuck at the separator surface (anode facing side) at 4.5 V, whereas the separator from the 4.3 V cell shows no visible deposits. Figure S2 shows SEM/EDX images of the black spots at the separator from the 4.5 V cell, clearly indicating that Li metal dendrites are stuck at the surface of the separator (anode facing side). The EDX analysis indicates electrolyte decomposition products (phosphorous and fluorine species) at the Li metal dendrites, whereas only minor amounts of Mn could be detected at the separator (Figure S2e–h).

**Figure 3:**
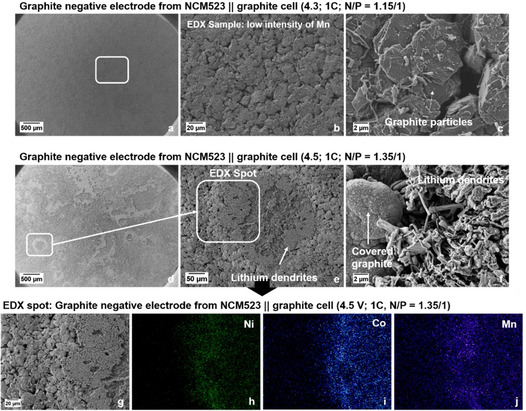
SEM/EDX analysis of graphite negative electrodes after 100 cycles in NCM523∥ graphite full cells (coin cells, two‐electrode configuration), as shown in Figure 2. (a‐c) SEM images of the graphite electrodes after cycling at 4.3 V; (d–f) SEM images of the graphite electrodes after cycling at 4.5 V. (g) Enlarged area of the SEM image in (e). (h–j) EDX elemental mappings of Ni (h), Co (i) and Mn (j) of the selected area of (g).

To examine the observed thick deposits at 4.5 V, especially in regard of formed Li metal dendrites, the anodes at 4.3 V and 4.5 V were systematically analyzed by means of SEM. According to the SEM images of the anode from 4.3 V cells (Figure 3a–c) no obvious thick deposits are apparent at the graphite anode surface. By taking a closer look at the graphite particles (Figure 3c) a small amount of Li metal dendrites (in the form of needles and very small white spots) are visible after 100 cycles at 1 C, even though the graphite particle surface is still visible and relatively smooth. Nearly no deposits or dendrites (spots/needles) can be observed for the 4.3 V cells using a higher N/P ratio of 1.50 : 1.00 (Figure S3b,c). The formation of Li metal was confirmed via ex situ solid‐state MAS NMR of the de‐lithiated graphite anodes after 100 charge‐discharge cycles. Whereas after cycling at 4.3 V no Li metal deposits could be detected (Figure S4), a characteristic ^7^Li signal at *δ*≈266 ppm was observed after cycling at 4.5 V, indicating the formation of microstructural Li metal (Figure S5).[^47^, ^48^] The resonance with high intensity at *δ*≈3 ppm can be attributed to significant SEI formation after cycling at 4.5 V.^[48]^ It should be noted that the absence of a significant SEI signal at 4.3 V is mainly caused by the reduced amount of SEI formed at this cell voltage and due to the subsequent washing procedure prior to the ^7^Li MAS NMR measurements.

In contrast, the graphite surface from the cell cycled at 4.5 V displays broad and thick deposits, appearing as large “islands” surrounded by a grey film (Figure 3d–f). These thick islands at the graphite anode can be identified as well‐formed Li metal dendrites (Figure 3f), whereas the grey film surrounding the dendrites is identified as highly covered graphite particles, covered with thick decomposition layers most likely through significant SEI formation and growth.^[46]^ Further SEM images can be found in Figure S6, showing the covered graphite particles and Li metal dendrites in different resolutions. It must be kept in mind that electrolyte decomposition and SEI formation will take place at both the graphite and Li metal surfaces, while the SEI components might be different. From EDX analyses, we found higher contents of fluorine and phosphorous at the spots of the Li metal dendrites compared to the graphite particles, indicating an increased decomposition of the LiPF_6_ salt at Li metal. As mentioned above, one major degradation mechanism of high‐voltage LIB cells can be attributed to the dissolution of transition metals (TMs) from the cathode material into the electrolyte, the consecutive migration through the electrolyte/separator and deposition at the anode surface, which results in further SEI growth.[^25^, ^26^, ^27^, ^30^, ^31^, ^32^] To investigate a possible correlation between Li metal dendrite formation and the highly covered graphite particles surrounding the Li dendrites (Figure 3d–f) in regard of TM deposition, EDX elemental mapping of various spots at the graphite anodes was performed.

Selected spots for the EDX analysis are depicted in Figure 3g–j, showing a significant accumulation of the three TMs nickel (Ni), cobalt (Co) and manganese (Mn; Figure 3h–j). Especially at the areas surrounding the Li metal dendrites, the highly covered graphite particles show the most intensive accumulation of these TMs. For further comparison, the EDX analysis of graphite anodes for the cells operated at 4.3 V is shown in Figure S7a. Whereas significant amounts of Mn, Ni and Co are found at the graphite surface at 4.5 V (Figure S7b), only small amounts of just Mn can be found when operated at 4.3 V.

Figure 2c and d shows the charge–discharge voltage profiles of NCM523∥ graphite full cells at 4.3 V and 4.5 V, respectively, each focusing on the 4^th^ cycle (two cycles after formation) and the 45^th^ cycle, that is, a few cycles before the rollover failure (Figure 2a). At 4.3 V, the difference of the voltage profiles between the 4^th^ and 45^th^ cycle is relatively small, only a discharge capacity loss of around 3 mAh g^−1^ can be noticed (Figure 2c). In comparison, major changes for the voltage profile from the 4^th^ to the 45^th^ cycle are apparent when cycled at 4.5 V, showing a compression of the profile (i. e., a reduction of charge and discharge capacity). A magnified view of the voltage profile (Figure 2d, inset) shows a significant increase of the voltage drop from 0.09 V to 0.32 V at 4.5 V in the 45^th^ cycle, whereas at 4.3 V the voltage drop is only 0.06 V and does not significantly change during cycling. This strong voltage drop in cycle 45 (4.5 V cells) indicates an increase of cell resistance, as it has been reported by others.[^18^, ^49^]

For consistency reasons, the SEM images shown in Figure 3 were recorded after 100 cycles. However, to exclude that the Li metal dendrites and accumulation of TMs occurs after the rollover failure (i. e., in the range between cycle 50 and 100), we additionally analyzed the graphite anode before the significant capacity decay, that is, after 40 cycles (Figure S8). Here, we also found the same results, that is, the formation of Li metal dendrites and the correlation to deposited TMs. Based on the observed Li metal dendrites at the graphite surface after cycling at 4.5 V (Figure 3d–f), the question raises if the significant voltage drop at 4.5 V (45^th^ cycle) is based on the resistance growth of NCM cathode or the graphite anode. From analysis of three‐electrode T‐cells, we found that the main part of cell impedance is caused by the NCM cathode (Figure S9). Further explanations can be found in the Supporting Information.

At this point, it should be addressed that the cathode (P) mass loadings of both the 4.3 V and the 4.5 V NCM523∥ graphite full cells were equal (12.2 mg cm^‐2^) and only the anode (N) capacity was adapted to achieve the same N/P ratio, whereas a broad range of N/P ratios was covered for the 4.3 V cells (N/P=1.15 / 1.00 to 1.50 / 1.00). This procedure is common to study cathode materials at different cutoff voltages and implies that at a cutoff of 4.5 V more lithium migrated from the cathode to the anode. Even though a higher lithium migration could be a likely reason for Li dendrite formation, we will exclude this possibility in the following chapter.

### 2.2 Impact of charge–discharge rate variation and cathode mass loading variation on cycling performance of NCM523∥ graphite full cells

Second, the charge–discharge rate was reduced from 1 C to 0.5 C to evaluate its impact on cycling performance during high‐voltage operation at 4.5 V, induced by the above discussed formation of thick and broadly spread electrolyte decomposition products and Li metal dendrites. The cycling performance of NCM523∥ graphite full cells (2.8‐4.5 V) at charge‐discharge rates of 0.5 C and 1 C is shown in Figure S10a. For both rates, the full cells show very similar capacity fading behavior, including the rollover failure after about 50 cycles, whereas the cell at 0.5 C delivers a slightly higher discharge capacity, which can be attributed to improved kinetics at lower rates.[^19^, ^50^] However, the rate reduction from 1 C to 0.5 C could not prevent the formation of thick and broadly spread deposits and Li metal dendrites at the anode surface, as shown in Figure S10b–e. Further explanations on the deposition morphology can be found in the Supporting Information.

**Figure 4:**
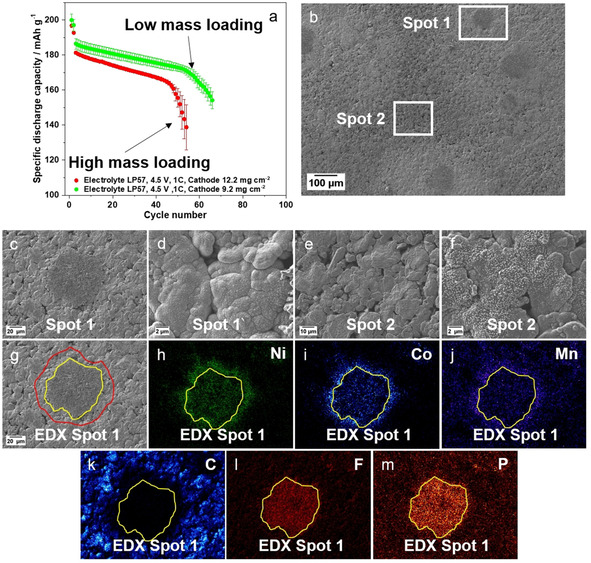
(a) Comparison of the charge–discharge cycling performance of NCM523∥ graphite full cells (coin cells, two‐electrode configuration) in a cell voltage range of 2.8–4.5 V (charge‐discharge rate: 1 C=190 mA g^−1^; N/P ratio=1.35 / 1.00) with NCM523 cathodes having a high mass loading (12.2 mg cm^−2^) and a low mass loading (9.2 mg cm^−2^). (b) SEM image of the graphite negative electrode after cycling (low mass loading cathode). (c–m) SEM images and EDX elemental mappings of the cycled graphite electrodes: (c) Spot 1 (dark grey dendrite spot); (d) spot 1 (highly covered graphite particles); (e,f) spot 2 (less covered graphite particles and no visible lithium dendrites); (g) selected dendrite island for EDX mapping (spot 1); (h–m) EDX elemental mappings of Ni (h), Co (i), Mn (j), carbon (k), fluorine (l), and phosphorus (m) for the selected area of (g).

Third, the cycling performance of NCM523∥ graphite full cells (4.5 V, 1 C) with different cathode mass loadings of 9.2 mg cm^−2^ (low mass loading) and of 12.2 mg cm^−2^ (high mass loading) was evaluated (Figure 4). The LIB cell operation at a fixed upper cell cutoff voltage at 4.3 V and at 4.5 V differs in the amount of charge [i. e., lithium (=Li^+^ and e^−^)] migrating from the cathode to the anode and vice versa during charge–discharge. In order to exclude that the higher amount of lithium migration in combination with the fast charge–discharge rate (i. e., 1 C) is not the reason for the formation of Li metal deposits, we also analyzed NCM cathodes with a lower mass loading. This cell setup resulted in a lower lithium migration from P to N, which is comparable to 4.3 V full cells.

Both full cells display a comparable cycling performance, showing rollover failure after around 50 cycles (Figure 4a). To investigate whether the reduction of the cathode mass loading from 12.2 mg cm^−2^ to 9.2 mg cm^‐2^ can prevent Li dendrite formation at the anode surface and to answer the question whether the rollover failure may be related to Li dendrite formation and/or the TM‐containing deposits or not, the cells with the low mass loading cathodes were disassembled according to the procedure discussed before and studied via SEM/EDX.

An enlarged region of the anode surface is depicted in Figure 4b, showing dark grey spots surrounded by a bright grey film. Similar spots have already observed with the high mass loading cathode (Figure 3d–f). One of the dark grey spots and a few micrometers of the adjacent surface grey film are defined as spot 1, whereas spot 2 is defined as a reference spot, where no visible surface deposition occurred. The dark grey spot was identified as Li dendrites (Figure 4c; highlighted with yellow marking around the spot in Figure 4g) and the grey film as highly covered graphite particles (Figure 4d; highlighted with red marking around the spot in Figure 4g) with thick decomposition layers (severe SEI growth). This observation is in line with the covered graphite particles observed before (Figure 3 and Figures S10–S12). Based on the EDX mapping of carbon, the graphite particles surrounding the Li dendrite spots are covered by a very thick surface layer, that no carbon species could be detected (Figure 4g,k). Similarly to the high mass loading cathodes, an accumulation of the three TMs (Ni, Co and Mn) as well as of Li dendrites is found at the graphite particle surfaces (Figure 4h–j). Additionally, EDX mapping of the elements phosphorus (P) and fluorine (F) shows their accumulation at the Li metal dendrites, which indicates strong LiPF_6_ conductive salt decomposition (Figure 4l,m).^[51]^

### 2.3 Impact of inhomogeneous Li metal deposition on cell performance

From the above findings, we postulate that there are two major cell failure modes in high‐voltage LIB cells: On one hand, there will be a significant capacity fading due to the ongoing consumption of active lithium, as a result of Li metal plating at the anode, whereas on the other hand – in the worst case – even a rollover failure is observed, which can be eventually linked to the generation of (micro‐) short circuits due to Li metal dendrites growing through the separator to the cathode. This postulation is verified by the following experiments.

**Figure 5:**
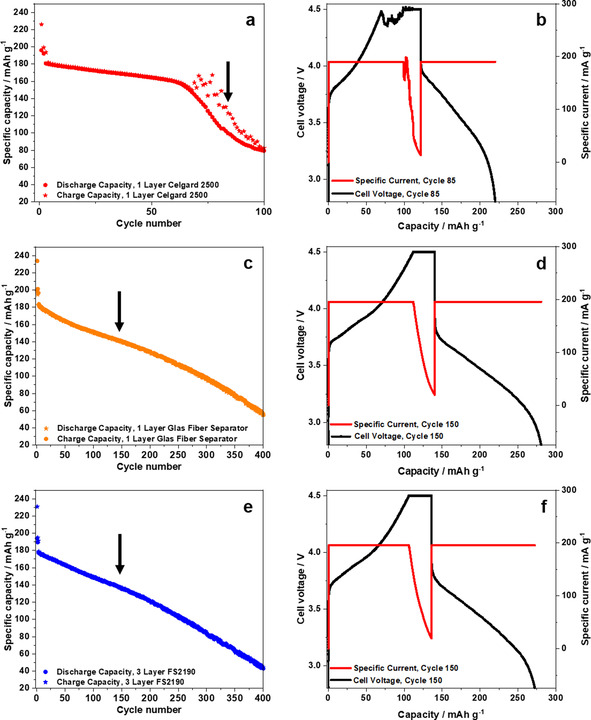
(a,c,e) Comparison of the charge‐discharge cycling performance of NCM523∥ graphite full cells (coin cells, two‐electrode configuration) in a cell voltage range of 2.8–4.5 V (cathode mass loading: 12.2 mg cm^−2^; charge‐discharge cycling rate: 1 C (= 190 mA g^−1^ at 4.5 V); N/P ratio=1.35 / 1.00) using different separators: (a,b) 1 layer Celgard 2500, (c,d) 1 layer glass fiber separator (Whatman) and (e,f) 3 layers of a polyolefin separator (Freudenberg FS2190). (b,d,f) Cell voltage and specific current profiles of selected cycles.

A strong indication of (micro‐) short circuits due to severe Li metal dendrite growth is given by the remarkable fluctuations of the charge capacity (Figure 5a) as well as the “cell voltage “noise” and specific current “noise” (Figure 5b) of the NCM523∥ graphite full cells operated at 4.5 V when using a relatively thin (≈25 μm) Celgard separator. In particular, Figure 5b displays the cell voltage and specific current curves of the 85^th^ cycle after the severe rollover failure, indicating voltage and current “noise” within the charge step, which is another hint for (micro‐) short circuits. Our hypothesis is also confirmed by the works of Homann et al. who observed a “voltage noise” in NCM622||Li metal cells based on a PEO‐based solid polymer electrolyte (SPE).[^52^, ^53^] They proved severe dendrite formation and penetration through the SPE to be the main source for “voltage noise” and sudden cell failure.^[52]^

Gilbert et al. observed similar fluctuations (referred to as “spikes”) in the charge capacities in NCM523∥ graphite full cells (3.0–4.4 V).^[26]^ However, they proposed that these charge capacity fluctuations result from fracture of NCM oxide particles, leading to enhanced dissolution of TMs from the cathode due to an increased electrochemical surface area. Further, they proposed that the gradual transformation of these newly exposed layered‐oxide surfaces to rock‐salt crystal structures results in increased cell impedance.^[26]^

From our results, we propose that the fluctuations are related to (micro‐) short circuits, which is supported by additional experiments: When exchanging the relatively thin Celgard separator by a thick glass fiber separator [thickness ≈680 μm (not compressed); Whatman; Figure 5c,d] or by three layers of a nonwoven polypropylene separator (thickness ≈230 μm (not compressed) for one layer; Freudenberg; Figure 5e,f), the charge capacity fluctuations and the voltage noise within the same cell setup cannot be observed anymore. In addition, no clear rollover failure can be observed, while still a dramatic capacity fading (within the first 100 cycles) is obvious, most likely indicating the proposed cell failure mechanism (I) in Figure 8e (see below). Thereby, the change of the separator will not significantly influence the phase transformation of the NCM cathode material. Further results, supporting our postulations, are shown in the Supporting Information (Figure S13). Future works may also focus on temperature changes of the cells, which can be another hint for (micro‐) short circuits.

**Figure 6:**
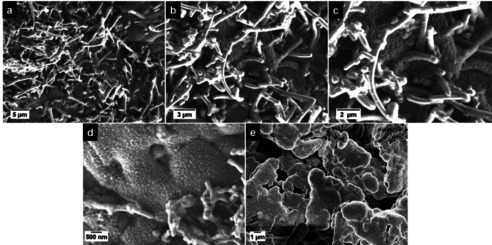
SEM images of a highly covered graphite particle and subsequent Li metal dendrite growth at the graphite particle surface. Graphite electrode obtained after charge–discharge cycling (100 cycles) in NCM523∥ graphite full cells (cell voltage range: 2.8–4.5 V; charge–discharge rate: 1 C=190 mA g^−1^; N/P ratio=1.35 / 1.00, cathode mass loading: 12.2 mg cm^−2^).

**Figure 7:**
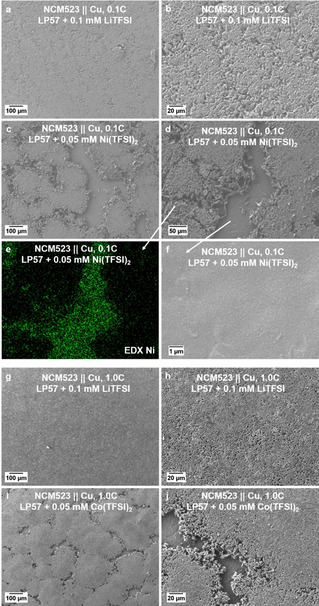
SEM images of copper foils obtained after the first charge to 4.2 V (at 0.1 C or 1 C) in NCM523∥ Cu cells using different electrolytes: (a,b) LP57+0.1 mM LiTFSI electrolyte at 0.1 C; (c,d,f) LP57+0.1 mM Ni(TFSI)_2_ electrolyte at 0.1 C; (g, h) LP57+0.1 mM LiTFSI electrolyte at 1 C; (i,j) LP57+0.05 mM Co(TFSI)_2_ at 1 C. Section (e) shows the EDX elemental mapping of Ni from (d).

**Figure 8:**
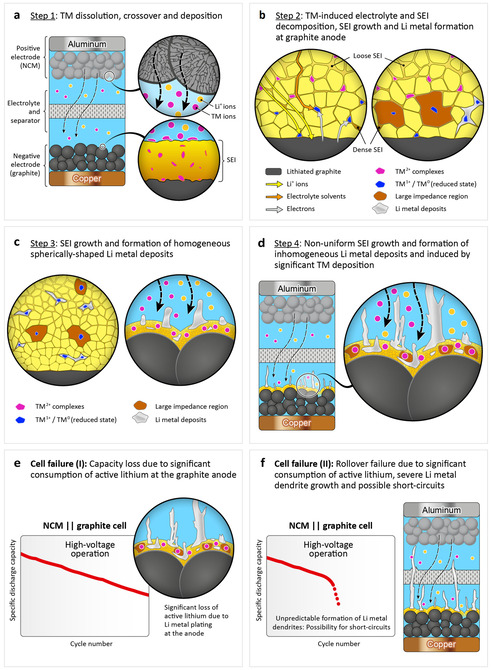
Schematic illustration of the major failure mechanisms in high‐voltage operated NCM∥ graphite LIB cells. (a) Step 1: TM dissolution from the NCM cathode, crossover and subsequent TM deposition at the graphite anode; (b) Step 2: TM‐induced electrolyte and SEI alteration and Li metal formation at the anode, according to an ion‐exchange model.[^37^, ^39^, ^64^] Reaction pathways for electrolyte reduction (orange arrows) and direct “underpotential deposition” of Li metal (yellow arrows) is catalyzed by TM species in the reduced state, close to the surface of lithiated graphite.;^[64]^ (c) Step 3: In an early state of charge–discharge cycling, the formation of homogeneous granular Li metal deposits is observed, likely involving a shielding mechanism;[^60^, ^61^] (d) Step 4: In a later state of charge–discharge cycling, formation of inhomogeneous Li metal deposits (i. e., dendrites) is observed, which is a result of further SEI alteration and growth, induced by severe TM deposition at the graphite anode; (e,f) Two different cell failure mechanisms are proposed: (e) steady capacity loss due to significant consumption of active lithium, as a result of Li metal plating at the anode and (f) “rollover failure” (rapid capacity drop) due to severe formation of Li metal dendrites and the possible formation of (micro‐) short circuits due to dendrites growing to the cathode.

The results show that the separator characteristics (e. g., thickness, fiber structure, porosity) have a major impact on the observed failure mechanism of high‐voltage LIB cells and its influence should not be neglected. However, this additional parameter makes it even more complicated to compare different studies from literature with respect to the TM dissolution/deposition mechanism and cell failure. For example, also the group of Gasteiger and co‐workers published important works on the behavior of TM dissolution in high‐voltage operated LIB full cells,[^25^, ^54^, ^55^, ^56^] however, a fair comparison to our works seems rather difficult due to the differences in cell setup. An example, in which no early high‐voltage induced rollover failure was observed, is given by the works of Laszczynski et al.^[54]^ and Jung et al.,^[55]^ studying NCM811∥ graphite full cells (3.0–4.6 V; 0.5 C charge–discharge rate; 25 °C) and NCM622∥ graphite full cells (3.0–4.6 V; 1 C charge–discharge rate; 25 °C), respectively. In both studies, the same electrolyte as in our work was used, that is, LP57 (1 M LiPF_6_ in EC/EMC 3 : 7), however, different separators were applied in the cell setup. Whereas we used a thin Celgard 2500 separator, Jung et al. and Laszczynski et al. used a rather thick glass fiber separator (Whatman) and three layers of separator (Celgard 2500/Whatman/Celgard 2500), respectively.[^54^, ^55^]

In summary, we would like to point out that various cell parameters, including the type and thickness of the separator can have a significant impact on the cell failure mechanism of high‐voltage LIB cells. Besides, we would also like to mention that certain types of separators, such as glass fiber separators (SiO_2_‐based), can display a significant reactivity towards the electrolyte components, as demonstrated by Markevich et al.^[57]^ Therefore, an additional impact on cycling performance, and this especially in high‐voltage LIB cells, can be expected.

### 2.4 Mechanistic elucidation of high‐voltage induced cell failure of NCM523∥ graphite full cells: Initial state of Li metal deposition

From the above results, we propose that the observed agglomerated spherically shaped spots at the graphite particle surface (Figures 3, 4, and S10) are initial Li metal nuclei, which further grow from the graphite particle surface to form dendrite‐like Li metal structures (Figure 6). This observation is in agreement with the work of Stark et al.^[58]^ They studied the Li metal dendrite growth on copper foil and observed very small hemispheric nuclei (ca. 100 nm range) in the beginning, which extrude over time to long dendrites. Similar Li metal deposits have been observed by Kim et al. at graphite anodes in NCM811∥ graphite full cells (2.8–4.2 V).^[59]^

We further propose that homogenous areal Li metal deposits will be formed at the graphite particle surfaces in an early state (Figure 6d), based on a possible electrostatic shield mechanism. The deposition of further TM species during ongoing cycling will most likely primarily take place at the Li metal spots, which can have a significant impact on the Li metal morphology. This assumption correlates well with the findings from Ding et al. and Yoon et al.,[^60^, ^61^] who showed that the addition of selected cations (such as Cs^+^) to the electrolyte significantly influenced the growth of Li metal dendrites at Li metal anodes. The Cs^+^ additive prevented the formation of long needle‐like dendrites and instead resulted in a very homogenous and dense Li metal deposition film, based on small spherically shaped Li metal depositions, which are also observed in our work. Yoon et al. pointed out that an effective self‐healing electrostatic shield mechanism is only possible for high concentrations of the cation (e. g., Cs^+^) in the electrolyte.^[61]^ Therefore, we propose that deposited TM species (especially Ni, Co species) have a similar effect in an early state of cycling, resulting in the formation of spherical Li metal nuclei. However, as the concentration of TM cations in the electrolyte might be too low for an effective shield mechanism, the formation of Li metal dendrites cannot be hindered during ongoing cycling (Figure 6). Furthermore, Mn deposits are known to hinder uniform SEI formation due to reaction with Li metal, thus, hindering uniform Li metal nucleation, as reported by Zhang et al. for Li metal batteries.^[62]^

To further verify the impact of the individual TM species on the Li metal deposition behavior and the appearance of Li metal deposits, the deposition behavior was evaluated in NCM523∥ Cu cells using different electrolytes: (I) LP57+0.1 mM LiTFSI at 0.1 C and 1 C, (II) LP57+0.1 mM Ni(TFSI)_2_ at 0.1 C, (III) LP57+0.05 mM Co(TFSI)_2_ at 1 C (Figure 7). After the first charge (at 0.1 C or 1 C) to a cutoff voltage of only 4.2 V (to avoid dissolution of all TM species), Li metal deposits on copper foil were studied via SEM and EDX. As shown in Figure 7, the use of LP57+0.1 mM LiTFSI electrolyte at 0.1 C and 1 C resulted in a uniform and dense Li metal deposition (Figure 7a,b,g,h), while the use of LP57+0.05 mM Ni(TFSI)_2_ resulted in a non‐uniform Li metal deposition at 0.1 C (Figure 7c–f). Additionally, the investigation of LP57+0.05 mM Co(TFSI)_2_ resulted in a similar non‐uniform Li metal deposition at 1 C (Figure 7i,j) compared to the Li metal deposition with Ni(TFSI)_2_ at 0.1 C.

Ni(TFSI)_2_ and Co(TFSI)_2_ were added to the electrolyte to demonstrate the impact of Ni or Co dissolution/deposition on the formed Li metal deposits on copper foil, showing an island‐like deposition. These dense Li metal island‐like deposits are partially grown together (Figure 7c,d,j) and are surrounded by relatively thin, homogenous films based of small spherically shaped deposits (Figure 7f), showing the highest accumulation of Ni for the Ni(TFSI)_2_‐based electrolyte (Figure 7e). When Mn(TFSI)_2_ was added to the electrolyte, similar observations can be found, as depicted in Figure S14. These observations are comparable to our results for the NCM523∥ graphite full cells at 4.5 V (see Figure 3) and further indicate that TMs such as Ni or Co species can significantly influence the process and morphology of Li metal deposition. In this context, Yuan et al. also reported that certain metal species (e. g., metal fluorides) can have a significant impact on the uniformity of Li metal deposition and dissolution in Li metal cells.^[63]^ The different impacts of various dissolved TMs on the morphology of Li metal deposits on Li metal has been also reported by Betz et al.^[40]^ Overall, these studies give a first indication of the significant impact of TMs on the homogeneity and uniformity of Li metal deposits, however, further studies are needed to clearly understand the impact of individual TMs and specific TM species.

### 2.5 Mechanism of high‐voltage induced cell failure of LIB cells caused by TM‐induced Li metal growth at the graphite anode: The worst‐case scenario

Our postulated correlation between deposited TMs at the graphite anode and deposited Li metal dendrites is well in accordance with previous studies.[^59^, ^64^] Li et al. demonstrated that notable Li metal microstructures were formed at the graphite anode surface in LiNi_0.61_Co_0.12_Mn_0.27_O_2_ ∥ graphite full cells (3.0–4.2 V; 1 C charge–discharge rate; 30 °C), which was promoted by before deposited TMs (mainly Mn).^[64]^ They found that the thickness of the Li metal deposits increased from approximately 24 nm (after 200 cycles) to 80 nm (after 3000 cycles) while a simultaneous increase in the Mn content at the graphite anode was noted. Most of the Mn species at the anode were found at the top surface of the SEI layer, which was not available for electron transfer due to its basically insulating nature, while only small amounts were detected in the interior SEI layer, close to the graphite surface. The TM‐promoted reduction of Li^+^ ions (i. e., Li metal formation and growth) was explained by an ion‐exchange (metathesis reaction) mechanism between Li^+^ ions and TM ions within the interior SEI layer, sufficiently close to lithiated graphite: Thereby, an electron transfer reaction can occur between LiC_*x*_ and e. g. TM^2+^, which can result in the formation of TM^1+^/TM^0^ species, thus promoting the deposition of Li metal and enhanced electrolyte decomposition.[^37^, ^39^, ^64^] It should be noted that their reference electrolyte system already included vinylene carbonate (VC) as electrolyte additive,^[64]^ which could already significantly slow down the cell failure (see chapter 2.6). Additionally, Kim et al. reported the formation of a non‐uniform and porous SEI layer and demonstrated the formation of Li metal dendrites at the anode in NCM811∥ graphite full cells (2.8–4.2 V; 45 °C) after 500 cycles caused by Ni deposits (Ni^0^ nanoparticles).^[59]^

The investigation of the oxidation state of the TMs in the SEI layer is still challenging, due to the complex nature of the SEI. Wandt et al. demonstrated that no metallic TMs were found at the graphite anode during cycling of NCM111∥ graphite cells (3.0–4.6 V; 20 °C), as the TMs (Ni, Co, Mn) were primarily present in the oxidation state 2+.^[65]^ However, in contrast to Ni and Co, Mn was also found as Mn^0^ on lithiated graphite by ex situ analysis.^[65]^ In this context, Solchenbach et al. proposed a mechanism for continuous SEI and electrolyte decomposition based on the reaction between SEI components [mainly lithium ethylenedicarbonate (LEDC)] and Mn^0^.^[66]^ Thereby, the re‐oxidation of the Mn^0^ to Mn^2+^ must be faster than the reduction of Mn^2+^ to Mn^0^, otherwise it should have been observed in the operando study by Wandt et al.^[65]^ In contrast, Kim et al. found metallic nickel at the graphite surface in NMC811∥ graphite full cells after cycling via XPS studies.^[59]^ Li et al. observed Li metal deposits, promoted by TM deposition, already at a rather low upper cell cutoff voltage of 4.2 V after 200 cycles.^[64]^ They further proposed that even more demanding conditions, including higher upper cutoff voltages (e. g., 4.4 V), fast charging conditions (e. g., 1.2 C), and elevated operating temperatures (e. g., 55 °C), which are not uncommon for EV applications, could significantly exacerbate the growth of Li metal deposits, promoted by crossover of TMs.^[64]^

Additionally, Gilbert et al. published a comprehensive study on the impact of TM deposits at the graphite anode,^[26]^ showing a strong capacity loss of around 37 % after 200 cycles in NCM523∥ graphite full cells (3.0–4.4 V, 1 C charge–discharge rate, 30 °C), whereas our cells only showed a capacity loss of approximately 13 % at 4.4 V and at 1 C (20 °C), as depicted in Figure S15. By increasing the upper cutoff voltage from 4.4 V to 4.45 V, they already observed a capacity loss of around 51 %, with an increased amount of TMs at the graphite anode after 200 cycles. They concluded that the main capacity loss could be attributed to trapped lithium in the SEI caused by the TMs’ parasitic reactions.^[26]^ In our work, we used an upper cutoff voltage 4.5 V, therefore, the impact of the TM dissolution/deposition on capacity fading should be even higher.

These few examples from literature indicate the high complexity of the investigation of TM deposition in high‐voltage LIB full cells and the unpredictable behavior of TM deposits at graphite anode, which can depend on various material, cell and electrochemical parameters. Further, it is rather challenging to compare results from different studies, as even moderate parameter changes can significantly influence cell performance and cell failure, such as N/P ratios, separator, electrolyte (additives) and material coatings.[^42^, ^67^]

The extreme capacity decay or – in the worst case – rapid rollover failure within the first 100 cycles in high‐voltage operated LIB full cells has been observed by different researchers.[^27^, ^68^, ^69^, ^70^, ^71^, ^72^, ^73^, ^74^] However, we did not find any tangible indicators with respect to the correlation between the rollover failure and TM‐induced Li metal deposition and growth at the anode, as the majority of research papers on high‐voltage operating LIB full cells primarily focuses on the investigation of the cathode itself and the CEI layer.

Our results demonstrate that Li metal dendrite growth caused by TM deposits leads to a rollover failure at early stages of cycling, thus being a serious problem at operation conditions leading to strong TM dissolution, e. g., the high‐voltage conditions. We believe that our work presents a highly challenging scenario, as we show that the deposited TMs can promote the formation of extremely thick Li metal deposits in NCM‐based full cells operated at high‐voltage. As the formation and growth of Li metal deposits is rather unpredictable and depends on various parameters,[^75^, ^76^, ^77^] it is highly important to gain further fundamental knowledge for the reasons and underlying mechanisms.

We postulate a possible failure mechanism for the high‐voltage NCM based full cells, as depicted in Figure 8. To explain the mechanism of decomposition layer growth at the graphite anode and subsequent formation of Li metal dendrites, influenced by TM crossover from the cathode during cycling, we would also like to refer to the comprehensive works and hypotheses from literature.[^26^, ^37^, ^39^, ^59^, ^64^, ^78^, ^79^, ^80^] Furthermore, we would like to point out the novelty of our work, that is, the severe formation of Li metal deposits at high‐voltage operation and the correlation to the observed rollover failure. In summary, the failure mechanism for the TM‐induced Li metal dendrite growth and subsequent rollover failure in LIBs cells operated at high‐voltage (e. g., 4.5 V) can be described by four major steps, as schematically illustrated in Figure 8:

1. In a first step, TM dissolution from the NCM cathode will take place, e. g., by attack of acidic species (such as HF) generated from electrolyte decomposition.[^37^, ^39^, ^64^] TM dissolution can result in NCM particle cracking during cycling and subsequently in the formation of newly generated particle surfaces,^[59]^ which can be further attacked by acidic electrolyte species. The dissolved TMs will be deposited at the graphite anode, however, we primarily observe Mn deposition at the beginning of charge–discharge cycling at high‐voltage (Figure 8a). The deposited TMs induce ongoing parasitic side reactions, that is, alteration of SEI components. This process can be catalyzed by TM species close to the lithiated graphite anode surface (TM^0^ and TM^1+^ in the reduced state), according to an ion‐exchange model.[^37^, ^39^, ^64^]
2. As a result of ongoing TM‐induced alteration of SEI components and SEI growth, regions of large impedance can be formed within the SEI which, in turn, impede or block Li^+^ diffusion within these SEI regions during charging (Figure 8b).^[64]^ The formation of first Li metal deposits is promoted by further SEI layer thickening at the anode and can be catalyzed by before deposited TM species (direct “underpotential deposition”).^[64]^ Furthermore, significant ongoing deposition of TM species (Mn, Ni, Co) at high‐voltage operation primarily takes place at or close by the formed Li metal deposits, as observed in this work.
3. In an early state, homogenous areal Li metal deposits (spherical nuclei) will be formed at the graphite particle surfaces based on a possible electrostatic shielding mechanism (Figure 8c).[^60^, ^61^] We propose that the deposited TM species (especially Ni, Co species) have a significant impact on the Li metal nuclei morphology in an early state of cycling. In contrast, Mn deposits are known to hinder uniform SEI formation due to reaction with Li metal, thus, hindering uniform Li metal nucleation.^[62]^
4. During ongoing charge–discharge cycling, further homogenous Li metal deposition cannot be longer guaranteed due to severe SEI breakage/growth and an ineffective shield mechanism, resulting in the formation of needle‐like Li metal deposits. This can be caused by gas evolution (e. g., CO_2_) during SEI decomposition,[^26^, ^37^] breakage of the SEI and cracking of the homogenous Li metal deposits, exposing newly formed surfaces, in combination with further TM deposition. Further Li metal deposits will preferably be formed at the needle‐like Li deposits and result in a dramatic rise of Li metal dendrite growth (in micrometer scale) with ongoing cycling (see Figure 6 and Figure 8d).

Finally, based on our findings, we postulate that there are two major cell failure modes in high‐voltage LIB cells: On one hand, there will be a significant capacity fading (I) due to the ongoing consumption of active lithium, as a result of Li metal plating at the anode (Figure 8e), whereas on the other hand – in the worst case – even a rollover failure (II) is observed, which can be eventually linked to the generation of (micro‐) short circuits due to Li metal dendrites growing to the cathode (Figure 8f).

### 2.6 Factors influencing the cycle life of high‐voltage LIB cells

#### 2.6.1 Impact of N/P capacity balancing ratio on the cycling performance of NCM523∥ graphite full cells

Another important factor which can influence the growth of Li dendrites and, thus, the cycling performance, is the anode to cathode capacity balancing ratio (N/P ratio).[^42^, ^81^, ^82^] In the previous chapters, a N/P ratio of 1.35 / 1.00 was used for full cells, while in this chapter the impact of an N/P ratio of 1.05 / 1.00 will be evaluated, and in particular, how it can influence the dendrite growth and cycle life of the NCM523∥ graphite full cells.

The relatively thick decomposition layers at the graphite anode particles, as shown in Figure 6 and Figure S6, induced by TM deposition, have also been observed by other researchers.[^27^, ^31^, ^46^] For example, Zheng et al. also observed these highly covered anode particles, combined with the high accumulation of the three TMs (Ni, Co and Mn) in NCM111∥ MCMB graphite full cells.^[27]^ To our knowledge, so far there are no publications about thick Li metal dendrite formation, induced in well capacity‐balanced high‐voltage LIB full cells (N/P ratio of ≥1.1 / 1), as reported here. In general, it is well‐known that the use of an N/P ratio >1 (capacity‐oversized anode) is mandatory to avoid anode potentials close to 0 V vs. Li|Li^+^ and, thus, to reduce the risk of Li metal plating at the anode in a LIB full cell setup.[^42^, ^82^] Dietz Rago et al. found a high accumulation of the three TMs (Ni, Co and Mn) at the surface of dendrites and surroundings in overcharged NCM523∥ graphite full cells.^[46]^ In their overcharge experiment, e. g., for a capacity‐balanced NCM523∥ graphite cell at 4.2 V, the cells were forced to higher cell voltages (up to 5 V) and, thus, higher cathode potentials, resulting in a higher de‐lithiation degree of the NCM‐based cathode. In turn, the N/P capacity balancing did not match anymore, resulting in an excess of lithium and in Li metal plating at the anode, which might cause a short circuit in the worst‐case.^[46]^ The observations by Dietz Rago et al. and the correlation between the formed Li metal dendrites and the accumulation of the TMs in the overcharged NCM523∥ graphite cells is similar to our observations of dendrite formation in capacity‐balanced NCM523∥ graphite full cells at 4.5 V (Figure 3d–f and FigureS10b–e). However, as a major difference, the anode in our works was capacity‐oversized by around 35 % (N/P=1.35 / 1.00) based on the practical capacity of around 190 mAh g^−1^ (4.5 V) for the NCM523 cathode and around 360 mAh g^−1^ for the graphite anode and, thus, should not result in an excess of lithium at the anode.

**Figure 9:**
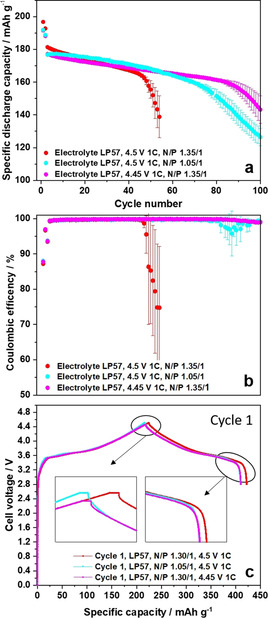
Comparison of the charge–discharge cycling performance of NCM523∥ graphite full cells (coin cells, two‐electrode configuration) in cell voltage ranges of 2.8–4.45 V (N/P=1.35 / 1.00) and 2.8–4.5 V (N/P=1.35 / 1.00 and 1.05 / 1.00). Cathode mass loading: 12.2 mg cm^−2^; charge–discharge cycling rate: 1 C [=185 mA g^−1^ at 4.45 V (N/P=1.35 / 1.00);=190 mA g^−1^ at 4.5 V (N/P=1.35 / 1.00);=190 mA g^−1^ at 4.5 V (N/P=1.05 / 1.00)]. (a) Discharge capacity and (b) Coulombic efficiency over cycling. (c) 1^st^ cycle charge‐discharge cell voltage profiles at 4.45 V (N/P 1.35 / 1.00), 4.5 V (N/P 1.35 / 1.00) and 4.5 V (N/P 1.05 / 1.00).

The cycling performance of full cells with N/P ratios of 1.35 / 1.00 and 1.05 / 1.00 is shown in Figure 9a. The first cycle practical capacities of the cells with the 5 % and 35 % oversized anodes are 190 mAh g^−1^ and 195 mAh g^−1^, respectively. A reduced capacity by 5 mAh g^−1^ for the less anode‐oversized cell (5 %, N/P ratio=1.05 / 1.00) indicates less lithium extraction from the cathode, which is a result of reduced cathode and anode potentials (i. e., both potentials are shifted to lower values).^[42]^ This capacity reduction in the 5 % anode‐oversized cell results in a significantly improved cycling performance, whereby the rollover failure is postponed to a higher cycle number, as well as into a more stable *C*_Eff_ (Figure 9b). To further demonstrate the significance and impact of small differences in the delithiation degree, that is, the cutoff potential^[42]^ of the cathode material on cycling performance, we cycled the NCM523∥ graphite full cells at an upper cutoff voltage of 4.45 V, however, with a 35 % oversized anode (Figure 9a). In this way, this cell is comparable to the 4.5 V cell using the 5 % oversized anode. It can be clearly seen that the 4.45 V cell (35 % oversized anode) has a similar discharge capacity compared the 4.5 V cells using the 5 % oversized anode. However, the cycle life is significantly improved by the minor reduction of the cutoff voltage to 4.45 V and, thus, by a slight reduction of the de‐lithiation degree of the cathode.

**Figure 10:**
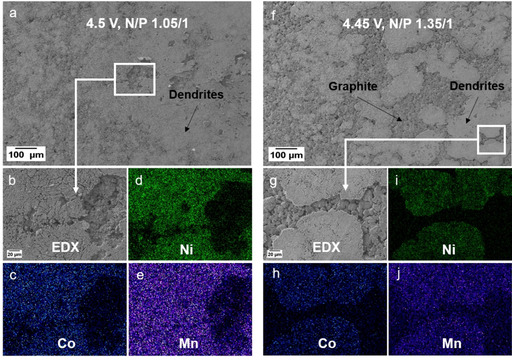
SEM and EDX elemental mapping analysis of the graphite negative electrode after cycling in NCM523∥ graphite full cells (see Figure 9) in cell voltage ranges of (a–e) 2.8–4.5 V (N/P=1.05 / 1.00) and (f–j) 2.8–4.45 V (N/P=1.35 / 1.00). (a) SEM image of the graphite anode for the cell operated at 2.8–4.5 V (N/P=1.05 / 1.00); (b–e) the corresponding EDX elemental mappings of Ni (d), Co (c), and Mn (e). (f) SEM image of the graphite anode for the cell operated at 2.8–4.45 V (N/P=1.35 / 1.00); (g‐j) the corresponding EDX elemental mappings of Ni (i), Co (h), and Mn (j).

Figure 9c shows the related charge–discharge cell voltage profiles of the NCM523∥ graphite full cells at 4.45 V (N/P=1.35 / 1.00), 4.5 V (N/P=1.35 / 1.00) and 4.5 V (N/P=1.05 / 1.00) upper cutoff voltages in the 1^st^ cycle. The 4.45 V (N/P=1.35 / 1.00) and the 4.5 V (N/P=1.05 / 1.00) cells result in very similar discharge capacities in the 1^st^ cycle and can thus explain the similar cycling behavior compared to the 4.5 V (N/P=1.35 / 1.00) cells. Especially the first charge curve of the 4.5 V (N/P=1.05 / 1.00) cell clearly shows that the cutoff voltage was reached earlier compared to the 4.5 V (N/P=1.35 / 1.00) cells. The related diminution of lithium extraction from the cathode for the 4.5 V (N/P=1.05 / 1.00) cells is in line with a decrease in discharge capacity. Overall, we suggest that the lower discharge capacity or rather the lower amount of extracted lithium from the cathode slows down the failure mechanism of the cells [4.5 V (N/P=1.05 / 1.00) and 4.45 V (N/P=1.35 / 1.00)], as observed by a shift of the rollover failure to higher cycle numbers (Figure 9a). According to the SEM images in Figure 10, showing the graphite anodes after cycling at 4.5 V (N/P 1.05 / 1.00) and 4.45 (N/P 1.35 / 1.00), a change in dendrite growth can be observed. The 4.5 V (N/P=1.05 / 1.00) cells (Figure 10a–e) and the 4.45 V (N/P 1.35 / 1.00) cells (Figure 10f–j) show a less localized, significantly broader distribution of the Li metal dendrites than the cells cycled at 4.5 V (N/P=1.35 / 1.00; Figure 3 and Figure S10). Thereby, dendrite “islands” are already visible for 4.5 V (N/P=1.05 / 1.00) cells, and they are “growing together” with ongoing cycling. However, according to EDX mapping of the three TMs (Ni, Co and Mn), an accumulation of TMs is present at the Li metal dendrites in the 4.5 V (N/P=1.05 / 1.00) cells and the 4.45 V (N/P=1.35 / 1.00) cells (Figure 10).

Both strategies, the slight reduction of the cell voltage to 4.45 V and the decrease in the anode oversizing, result in a similarly improved cycling performance. However, both approaches cannot prevent the formation of thick Li metal dendrites at the graphite anode as well as the accumulation of the TMs at the dendrite surfaces and their surroundings (Figure 10), in turn still inducing the rollover failure, which can only be postponed to higher cycle numbers.

### 2.7 Impact of NCM particle morphology: Single‐crystal vs. polycrystalline NCM materials

In this last chapter, we would like to present strategies to tackle the TM‐induced “rollover” failure in high‐voltage LIB cells and verify these strategies as proof‐of‐principle. In general, the intrinsic properties of the NCM cathode material can significantly reduce the tendency for TM dissolution and, thus, improve overall cell performance. In this respect, cathode material coatings or dopings (the latter are actually substitutions), as well as the design of a single‐crystal morphology are considered as suitable approaches to enhance the cathode material stability, allowing an enhanced performance at high cell voltages (≥4.5 V).[^6^, ^83^, ^84^, ^85^, ^86^]

**Figure 11:**
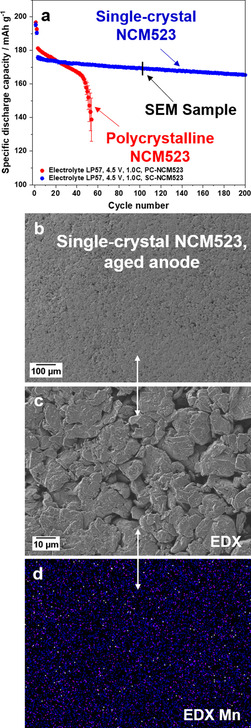
(a) Comparison of the charge–discharge cycling performance of NCM523∥ graphite full cells (coin cells, two‐electrode configuration) in a cell voltage range of 2.8–4.5 V (cathode mass loading: 12.2 mg cm^−2^; charge‐discharge rate: 1 C=190 mA g^−1^; N/P ratio=1.35 / 1.00) using either polycrystalline NCM523 and single‐crystal NCM523 cathode materials. (b,c) SEM images of the cycled graphite anodes of the single‐crystal NCM523∥ graphite full cells. (d) EDX elemental mapping of the cycled graphite anode (Mn).

Whereas polycrystalline NCM523 materials could not hinder the early rollover failure in high‐voltage NCM523∥ graphite full cells, we like to verify the proposed beneficial impact of a single‐crystal NCM523 material in NCM523∥ graphite full cells. As shown in Figure 11a, the cells using the single‐crystal NCM523 material with the same graphite anode as for the polycrystalline NCM523 display no rollover failure as well as a significantly increased capacity retention within 200 charge–discharge cycles. Additionally, according to SEM (Figure 11b,c), no (thick) decomposition layers and in an enlarged view also no dendrites can be observed, whereas the cell based on polycrystalline NCM523 already shows small amounts of manganese and small dendrites at 4.3 V (Figure 3a–c). Nevertheless, minor amounts of Mn can be found at the graphite surface after 100 cycles, as shown by EDX elemental mapping (Figure 11d). According to ICP‐OES analyses of the cycled graphite anodes after 200 cycles, a high content of Ni (0.05 wt %) and Mn (0.02 wt %) was found for polycrystalline NCM523‐based cells, while no detectable amounts of Ni and Mn were found for the single‐crystal NCM523‐based cells.

In this context, Fan et al. reported that no particle cracks were observed for single‐crystal NCM811 particles compared to polycrystalline NCM811 particles after cycling in NCM811‐based cells.^[86]^ Thus, we assume that enhanced material stability is one of the main reasons for the significantly improved performance of single‐crystal NCM materials in high‐voltage cells. As no or less cracks are formed, less new surface area is generated, thus, less TMs should be dissolved due to less reactive interfaces towards the electrolyte. Additionally, the work of Li et al. confirmed the excellent cycling stability of NCM523 single‐crystals in LIB full cells.^[83]^ From our results, we also believe that the increased surface area due to ongoing NCM particle cracking is a major reason for enhanced TM dissolution, thus, resulting in increased TM‐induced Li metal plating at the anode.

The significant impact of the various material/electrode/cell/operation parameters, including the type of NCM material, must be kept in mind when comparing literature results in terms of cell performance.[^42^, ^67^] As shown above, a significant capacity drop of evaluated NCM523∥ graphite full cells was already observed at 4.45 V within 100 cycles. In contrast, Thompson et al. demonstrated that is possible to cycle NCM523∥ graphite full cells under aggressive conditions (2.8–4.4 V; 0.33 C charge–discharge rate; 55 °C) up to 750 cycles without any dramatic cell failure and noted a capacity loss of only about 10 %.^[84]^ However, the used electrolyte also included 2 wt % fluoroethylene carbonate (FEC) and 1 wt % 1,3,2‐dioxathiolane‐2,2‐dioxide (DTD) as additives.^[84]^ In contrast to Gilbert et al.,^[26]^ who cycled at 3.0–4.4 V and 30 °C, Thompson et al. found less TMs at the aged graphite anode. In our work, we observed the rollover failure and a high accumulation of the TMs at the anode surface, like Gilbert et al. The major difference between these studies is not only the electrolyte, but particularly the used NCM material: Whereas Thompson et al.^[84]^ studied the long‐term cycling performance using single‐crystal NCM523 material, our work and the work of Gilbert et al.^[26]^ used polycrystalline NCM523 particles. The use of single‐crystals in the high‐voltage operation full cells significantly influences cell performance, as no rollover within 200 cycles was observed at 4.5 V and 1 C (Figure 11) and only a loss of around 10 mAh g^−1^ was detected, displaying a capacity retention of 95 % (based on first cycle after the formation). The analysis of the aged anode of the single‐crystal NCM523∥ graphite full cells showed that no Ni and Co could be detected by EDX and no Li metal dendrite islands appeared after 100 cycles. Moreover, the rollover was effectively prevented at 4.5 V and 1 C, even though no electrolyte additives were used (pure LP57 electrolyte). These results are another hint for the proposed cell failure mechanism (Figure 8), that is, that the formation of thick Li metal deposits in combination with TM deposits are a major reason for the rollover cell failure of high‐voltage LIB cells.

The modification of the electrolyte, e. g., by use of suitable electrolyte additives, is another major strategy to address the high‐voltage cell failure. For example, Wu et al. did not observe an early rollover failure within 100 cycles for NCM622∥ graphite full cells (2.75–4.5 V; 0.5 C charge‐discharge rate; 20 °C).^[87]^ A major difference compared to our study was that their used reference electrolyte system already included 2 wt % vinylene carbonate (VC) in LP57.^[87]^ In order to verify the beneficial impact of VC in our system, we also evaluated NCM523∥ graphite full cells (Figure S16) and could observe that the severe rollover failure is prevented by adding 2 wt % VC, while detecting a comparable capacity retention as found by Wu et al.^[87]^ Therefore, the use of electrolyte additives in combination with suitable NCM material modifications, such as single‐crystal NCM materials, is a good strategy to improve the cycle life of high‐voltage LIB cells.

**Figure 12:**
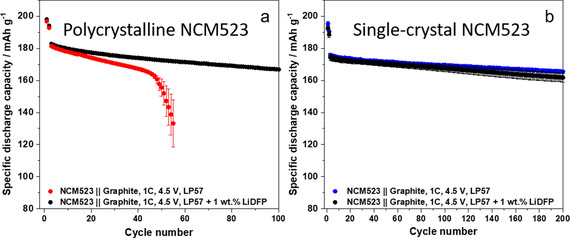
Comparison of the charge–discharge cycling performance of NCM523∥ graphite full cells (coin cells, two‐electrode configuration) in a cell voltage range of 2.8–4.5 V (cathode mass loading: 12.2 mg cm^−2^; charge‐discharge rate: 1 C=190 mA g^−1^; N/P ratio=1.35 / 1.00) using either (a) polycrystalline NCM523 or (b) single‐crystal NCM523 cathode materials. Two different electrolyte systems are used: LP57 (pure) and LP57+1 wt.% LiDFP.

As no significant cell failure occurs in single‐crystal NCM523‐based cells (Figure 11a), the question arises whether electrolyte additives can have a similar impact as observed in cells based on polycrystalline NCM materials. We postulate that electrolyte additives only play a minor role in LIB full cells using single‐crystal NCM cathodes compared to polycrystalline cathodes. For example, the electrolyte additive lithium difluorophosphate (LiDFP; 1 wt %) suppressed effectively the rollover in polycrystalline NCM523∥ graphite full cells with a capacity retention of 95 % after 100 cycles (Figure 12a). However, in single‐crystal NCM523∥ graphite full cells, the addition of 1 wt % LiDFP only had a negligible effect on the cell performance at 20 °C and 4.5 V within 200 cycles (Figure 12b).

The analysis of single‐crystal or differently modified NCM523 materials in high‐voltage LIB cells opens a completely new field and will be discussed in upcoming works with respect to TM dissolution and the impact on the degradation mechanism of high‐voltage LIB cells. This initial proof‐of‐principle study clearly points out that the modification and design of cathode materials can play a highly important role in inhibiting TM dissolution and, thus, suppressing the rollover failure of high‐voltage LIB cells. In addition, further systematic studies of novel electrolyte formulations, in particular additives, are needed to evaluate the impact on the long‐term cycling performance at high‐voltage conditions.

## 3 Conclusion

Herein, we have elucidated the severe impact of transition metal (TM) dissolution from the NCM523 cathode on the degradation mechanism of high‐voltage NCM523∥ graphite full cells, that is, on the rapid capacity fading also known as “rollover” failure. Various aspects influencing the performance of high‐voltage NCM523∥ graphite cells have been systematically evaluated, including the impact of (a) the upper cutoff voltage, (b) the charge–discharge rate, (c) the cathode mass loading, and (d) the N/P capacity balancing ratio.

Although a stable cycling performance was observed at 4.3 V, a strong capacity fading, and a rollover failure was already noted after around 50 cycles when increasing the upper cutoff voltage to 4.5 V. Such an early rollover failure in high‐voltage LIB cells has also been observed by various other researchers, however, the underlying mechanism was not completely understood so far.

The graphite anodes cycled at 4.3 V only showed minor amounts of Mn (most likely incorporated in the SEI layer), whereas full cells operated at 4.5 V showed broadly spread deposits at the anode surface, that is, significantly covered graphite anode particles (by thick deposits/decomposition layers) and Li metal dendrite formation (spots and needles) after cycling. These deposits and the spots near the Li metal dendrites could be correlated with the deposition of TMs (Ni, Co and Mn), whereas for spots at the anode surfaces with less deposits and no observable Li dendrites, just Mn and no Ni or Co elements could be detected. Therefore, we postulate that the dissolution of the different TMs (Ni, Co and Mn) from the cathode and their deposition at the graphite anode results in severe electrolyte and SEI decomposition and non‐uniform SEI growth, which is significantly promoted by deposited TM species. In turn, these parasitic processes promote the formation of Li metal deposits, which can initially grow homogeneously, whereas needle‐like (dendrite) structures are also formed upon further charge–discharge cycling. Overall, we postulate that a main reason for the dramatic capacity fading in high‐voltage LIB cells is the ongoing consumption of active lithium at the anode, because of Li metal plating at graphite. In the worst‐case scenario even the “rollover” failure mechanism will be observed, which can be most likely linked to the generation of (micro‐) short circuits from dendrites growing through the separator to the cathode. These postulations have been verified by various experiments, e. g., using different separator materials within the same cell setup.

Furthermore, we found a stable cycling performance of a single‐crystal NCM523 material with (almost) no deposited TMs and no Li dendrites. This confirms the hypothesis that the main degradation mechanism of high‐voltage LIB full cells is induced by the dissolution/deposition of TMs and their incorporation into the SEI layer at the anode, which further induces SEI growth and Li metal deposition. We demonstrated that a fair comparison of literature results with respect to high‐voltage induced rollover and cell failure is rather challenging, due to various parameters (active materials, separator, electrolyte, N/P ratio, etc.) influencing the cell performance and aging mechanisms. Whereas some researchers reported the rollover failure in their cell setup, others under different experimental conditions only found strong capacity fading but no early rollover failure, which can be a result of minor changes of the used materials and cell components or variations within the cell setup and/or cycling conditions. For example, we also demonstrated that already small changes in the cell setup, for example the N/P ratio, can significantly enhance the cycle life, which in turn makes it very complicated to compare results among different research groups and publications.

This study clearly reveals that suppression of cross‐talk phenomena in NCM∥ graphite full cells operating at high‐voltage is of utmost importance for achieving a high cycling stability. However, at this point the underlying mechanism of TM deposition at the anode and the impact of the individual TMs (Mn, Co, Ni) on the degradation (i. e., SEI degradation and growth and Li metal plating at the anode) and subsequent rollover failure in high‐voltage full cells is not completely clear yet. We consider that all three TMs interact in different negative manners on the degradation of the anode SEI layer, however, further comprehensive studies are mandatory to prove this assumption.

## Conflict of interest

The authors declare no conflict of interest.

## Supporting information

As a service to our authors and readers, this journal provides supporting information supplied by the authors. Such materials are peer reviewed and may be re‐organized for online delivery, but are not copy‐edited or typeset. Technical support issues arising from supporting information (other than missing files) should be addressed to the authors.

Supplementary
