# Supplementary material for: Exploiting the Degradation Mechanism of NCM523 ∥ Graphite Lithium‐Ion Full Cells Operated at High Voltage
Source: ChemSusChem. 2020 Nov 10;14(2):595–613. doi: 10.1002/cssc.202002113 (PMC7894331; doi:10.1002/cssc.202002113)
Supplement: Supplementary file 1 — Supplementary [file CSSC-14-595-s001.pdf]

ChemSusChem

Supporting Information

Exploiting the Degradation Mechanism of NCM523 ||Graphite Lithium-Ion Full Cells Operated at High Voltage

Sven Klein, Peer Bärmann, Thomas Beuse, Kristina Borzutzki, Joop Enno Frerichs, Johannes Kasnatscheew, Martin Winter,* and Tobias Placke© 2020 The Authors. ChemSusChem published by Wiley-VCH GmbH. This is an open access article under the terms of the Creative Commons Attribution License, which permits use, distribution and reproduction in any medium, provided the original work is properly cited.

Supporting Information

Exploiting the Degradation Mechanism of NCM523 || Graphite Lithium Ion Full-Cells Operated at High Voltage

Sven Klein[1], Peer Bärmann[1], Thomas Beuse[1], Kristina Borzutzki[2], Joop Enno Frerichs[3], Johannes Kasnatscheew[2], Martin Winter[1,2,*], Tobias Placke[1,*]

[1] University of Münster, MEET Battery Research Center, Institute of Physical Chemistry, Corrensstr. 46, 48149 Münster, Germany

[2] Helmholtz Institute Münster, IEK-12, Forschungszentrum Jülich GmbH, Corrensstr. 46, 48149 Münster, Germany

[3] University of Münster, Institute of Physical Chemistry, Corrensstr. 30, 48149 Münster, Germany

<u>*Corresponding authors</u>:
Dr. Tobias Placke (tobias.placke@uni-muenster.de)
Prof. Dr. Martin Winter (m.winter@fz-juelich.de)

Experimental

Table S1. Positive and negative electrode compositions and characteristics.

Electrode Properties	Negative Electrode	Positive Electrode
Composition	95.0 wt.% graphite, 1.5 wt.% SBR, 3 wt.% Na-CMC, 0.5 wt.% carbon black	95.0 wt.% NCM523, 3.0 wt.% PVdF, 2.0 wt.% carbon black
Processing solvent	Water	NMP
Electrode mass loading	(a) 8.8 mg cm^{-2}<br>(b) 6.9 mg cm^{-2}	(c) 9.2 mg cm^{-2}<br>(d) 12.2 mg cm^{-2}
Electrode areal capacity	(a) 3.0 mAh cm^{-2}<br>(b) 2.3 mAh cm^{-}	(c) 1.7 mAh cm^{-2} (4.5 V)<br>(d) 2.2 mAh cm^{-2} (4.5 V)<br>2.0 mAh cm^{-2} (4.3 V)
Electrode diameter in two-electrode coin cells	Ø15 mm	Ø14 mm
Electrode diameter in three-electrode T-cells	Ø12 mm	Ø12 mm
N/P ratio<br>(Anode || Cathode)	**Chapter 2.1: "Impact of the Upper Cut-Off Voltage" (Figure 2)**<br>4.5 V → 1.35/1.00 (=3.0 || 2.2 mAh cm^{-2})<br>4.3 V → 1.50/1.00 (=3.0 || 2.0 mAh cm^{-2})<br>4.3 V → 1.15/1.00 (=2.3 || 2.0 mAh cm^{-2})<br>**Chapter 2.2: "Impact of Charge/Discharge Rate Variation and Cathode Mass Loading Variation" (Figure S10)**<br>4.5 V → 1.35/1.00 (=3.0 || 2.2 mAh cm^{-2})<br>**Chapter 2.2: "Impact of Charge/Discharge Rate Variation and Cathode Mass Loading Variation" (Figure 4)**<br>4.5 V → 1.35/1.00 (=3.0 || 2.2 mAh cm^{-2})<br>4.5 V → 1.35/1.00 (=2.3 || 1.7 mAh cm^{-2})<br>**Chapter 2.6.1: "Factors Influencing the Cycle Life of High-Voltage LIB Cells" (Figure 9)**<br>4.5 V → 1.35/1.00 (=3.0 || 2.2 mAh cm^{-2})<br>4.5 V → 1.35/1.00 (=2.3 || 1.7 mAh cm^{-2})<br>**Chapter 2.6.2 "Factors Influencing the Cycle Life of High-Voltage LIB Cells" (Figure 11)**<br>4.5 V → 1.35/1.00 (=3.0 || 2.2 mAh cm^{-2})<br>4.5 V → 1.05/1.00 (=2.3 || 2.2 mAh cm^{-2})	
Current collector	Copper foil (10 µm)	Aluminum foil (15 µm)
Porosity	30 %	30 %

Table S2. Cell characteristics and electrochemical data of NCM523 || graphite full-cells.

**(a) cathode mass loading**<br>**(b) charge/ discharge rate**<br>**(c) N/P ratio**	**Cell voltage range / V**	**Specific current at 1C / mA g^{-1}**	**Coulombic efficiency in 1st cycle / %**	**Capacity in 4th cycle / mAh g^{-1}**	**Capacity in 45th cycle / mAh g^{-1}**
**Chapter 2.1: "Impact of the Upper Cut-Off Voltage" (Figure 2)**<br>(a) 12.2 mg cm^{-2}<br>(b) 1C<br>(c) 1.35/1.00	2.8-4.3	170	89	162	159
**Chapter 2.1: "Impact of the Upper Cut-Off Voltage" (Figure 2)**<br>(a) 12.2 mg cm^{-2}<br>(b) 1C<br>(c) 1.35/1.00	2.8-4.5	190	87	180	164
**Chapter 2.2: "Impact of Charge/ Discharge Rate Variation and Cathode Mass Loading Variation" (Figure S10)**<br>(a) 12.2 mg cm^{-2}<br>(b) 0.5C<br>(c) 1.35/1.00	2.8-4.5	190<br>(0.5C = 95 mA g^{-1})	87	185	171
**Chapter 2.2: "Impact of Charge/ Discharge Rate Variation and Cathode Mass Loading Variation" (Figure 4)**<br>(a) 9.2 mg cm^{-2}<br>(b) 1C<br>(c) 1.35/1.00	2.8-4.5	190	85	184	173
**Chapter 2.6.1: "Factors Influencing the Cycle Life of High-Voltage LIB Cells" (Figure 9)**<br>(a) 12.2 mg cm^{-2}<br>(b) 1C<br>(c) 1.05/1.00	2.8-4.5	190	88	177	169
**Chapter 2.6.1: "Factors Influencing the Cycle Life of High-Voltage LIB Cells" (Figure 9)**<br>(a) 12.2 mg cm^{-2}<br>(b) 1C<br>(c) 1.35/1.00	2.8-4.45	185	88	177	167
**Chapter 2.6.2: "Factors Influencing the Cycle Life of High-Voltage LIB Cells" (single crystal NCM523; Figure 11)**<br>(a) 12.2 mg cm^{-2}<br>(b) 1C<br>(c) 1.35/1.00	2.8-4.5	190	86	175	172

ICP-OES analysis

ICP-OES measurements were performed using an ARCOS (Spectro Analytical Instruments GmbH, Kleve, Germany) with an axial positioned plasma torch. For analysis, the following emission lines were observed: 341.476 nm for nickel, 257.611 nm for manganese and 228.616 nm for cobalt. All other parameters and preparations were applied according to Vortmann and Evertz et al.[1, 2]

Further results of the chapter 2.1: "Impact of the Upper Cut-Off Voltage on the Cycling Performance of NCM523 || Graphite Full-Cells"

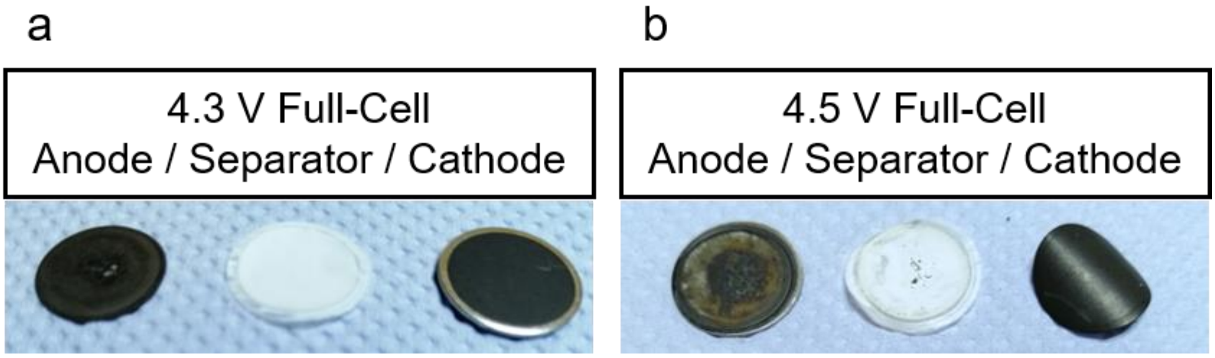

Figure S1. Photographs of the extracted electrodes and separators of the aged NCM523 || graphite full-cells after cycling (100 cycles) in a cell voltage range of a) 2.8-4.3 V and b) 2.8-4.5 V.

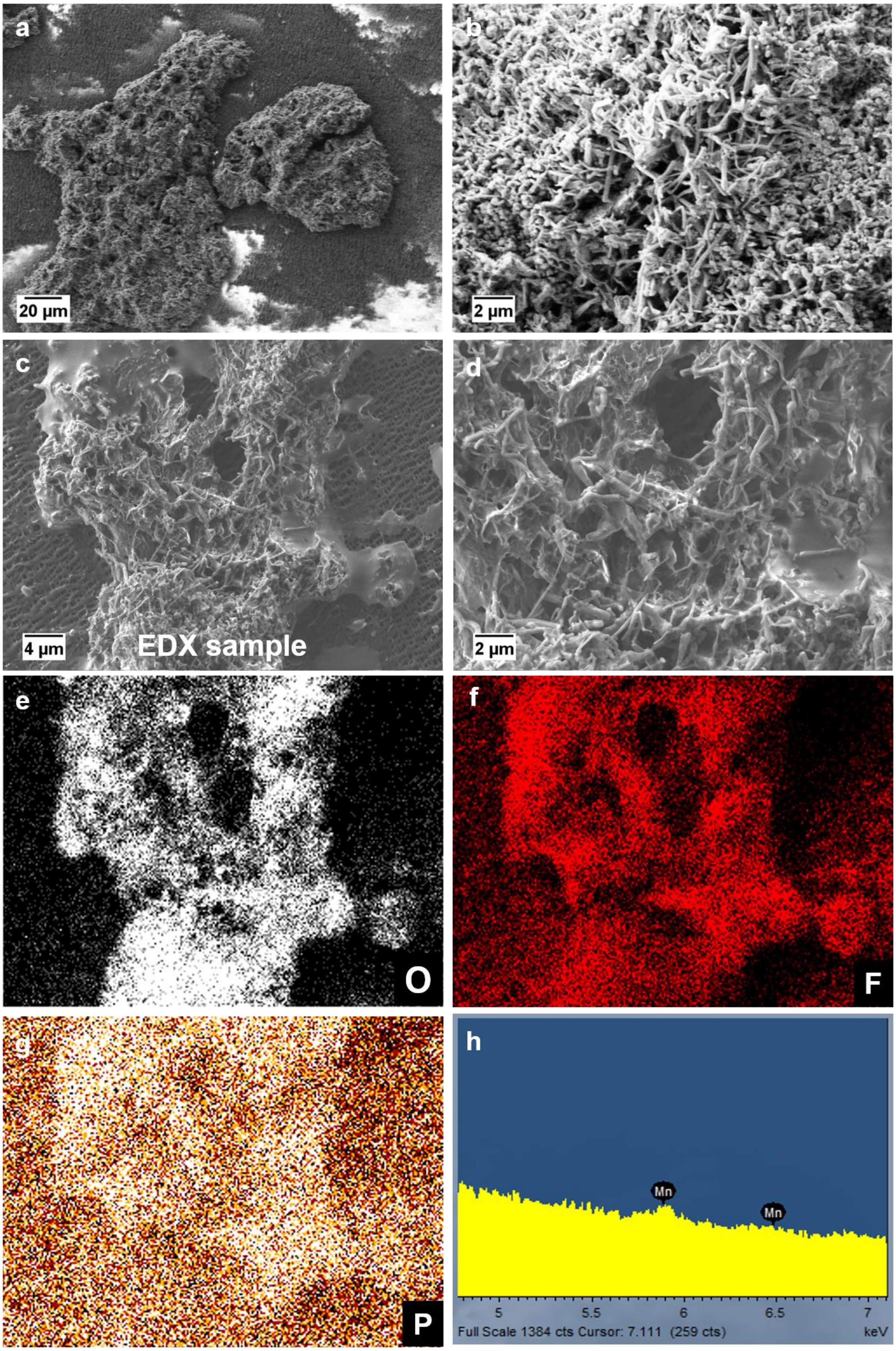

Figure S2. (a-d) SEM images of the extracted separators of the aged NCM523 || graphite full-cells after cycling (100 cycles) in a cell voltage range of 2.8-4.5 V, showing Li metal dendrites stuck at the separator surface (anode facing side). (e-h) EDX elemental mappings of O (e), F (f), P (g) and Mn (h) from the SEM image in (c).

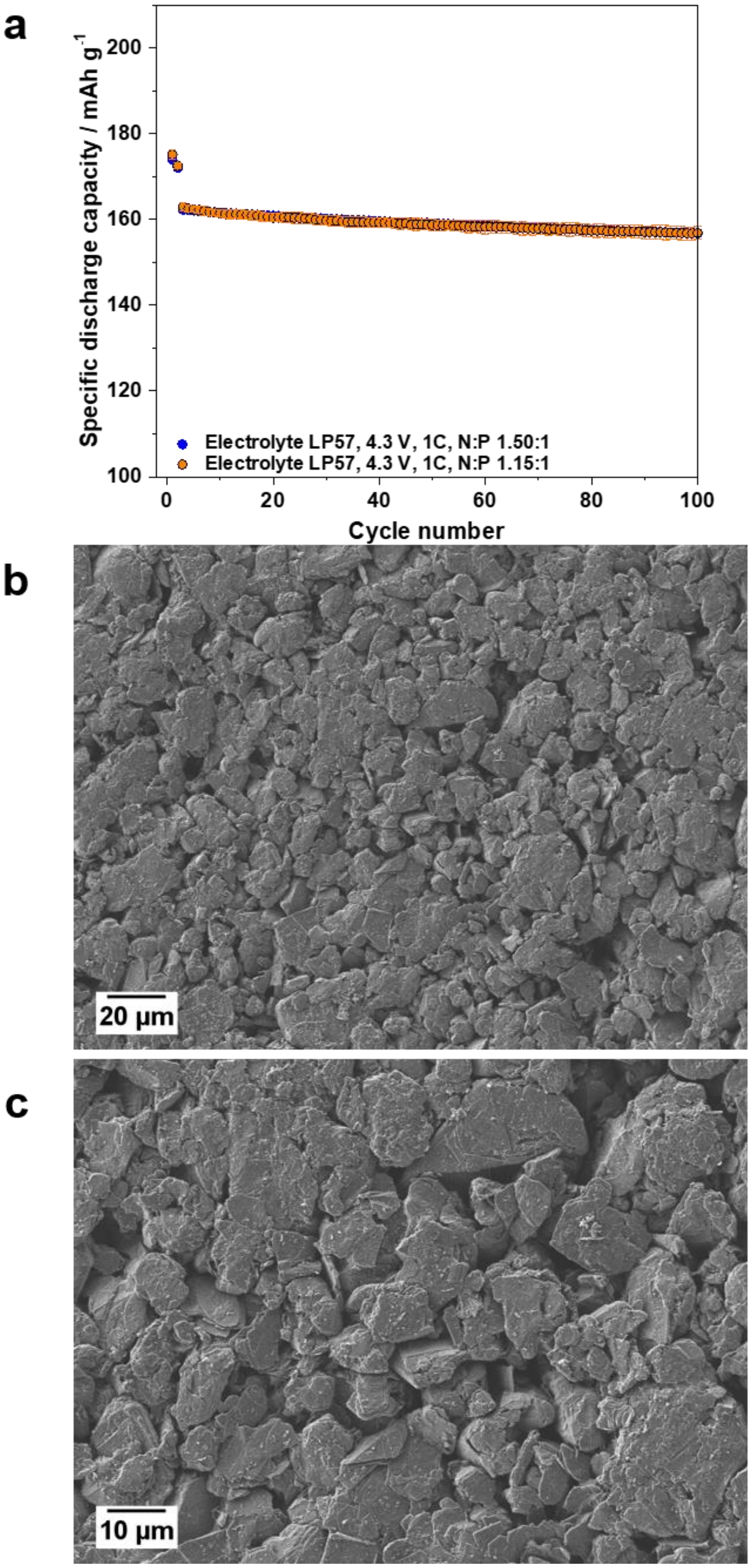

Figure S3. Charge/discharge cycling performance of NCM523 || graphite full-cells (coin cells, two-electrode configuration, (a) in the cell voltage range of 2.8 - 4.3 V (cathode mass loading: 12.2 mg cm^{-2}; charge/discharge cycling rate: 1C (= 170 mA g^{-1}); N/P ratios = 1.15/1.00 and 1.50/1.00) and SEM analysis of the graphite negative electrode after cycling (b, d).

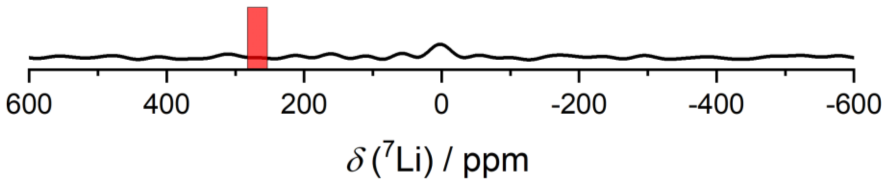

Figure S4. Ex situ ^{7}Li MAS NMR spectra of the de-lithiated graphite anode (after 100 cycles) using an upper cell cut-off voltage of 4.3 V (see **Figure 2**) recorded at 4.70 T (200 MHz) under 25 kHz MAS condition. The chemical shift region of microstructural Li metal is highlighted by a red insert.

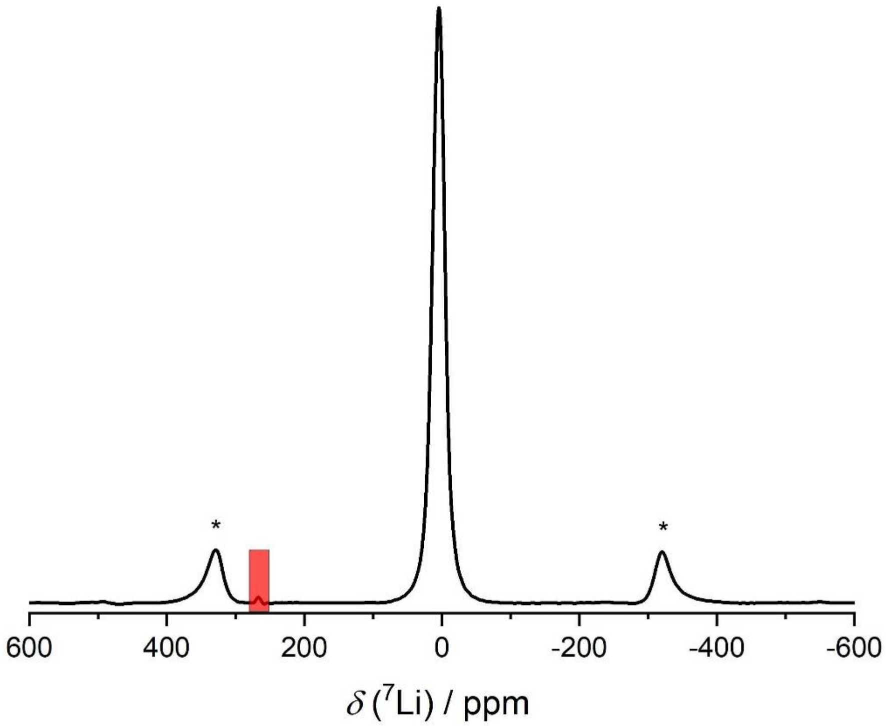

Figure S5. Ex situ ^{7}Li MAS NMR spectra of de-lithiated graphite anode (after 100 cycles) using an upper cell cut-off voltage of 4.5 V (see **Figure 2**) recorded at 4.70 T (200 MHz) under 25 kHz MAS condition. The chemical shift region of microstructural Li metal is highlighted by a red insert.

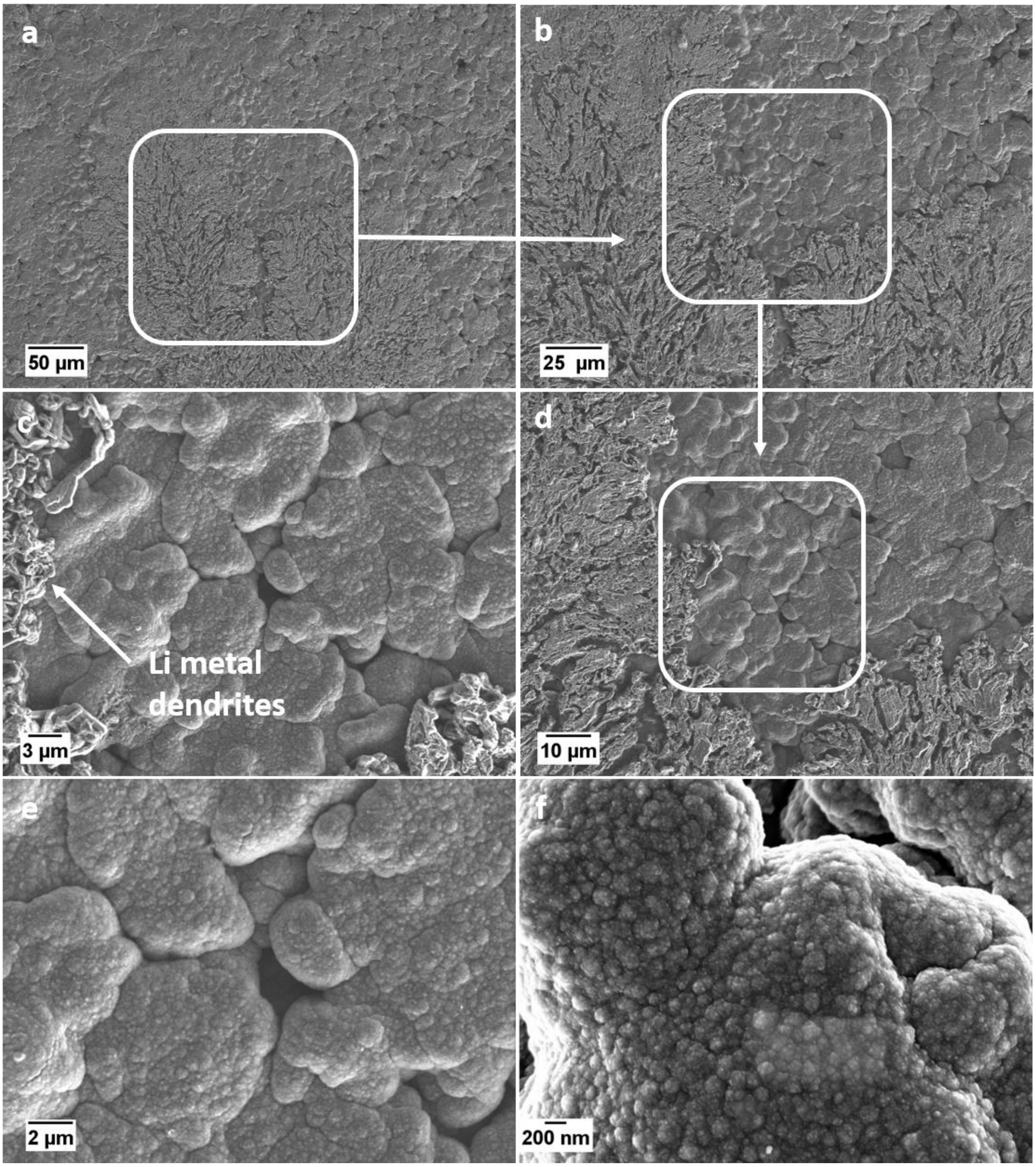

Figure S6. SEM images of the cycled graphite electrode surface after 100 cycles, obtained from NCM523 || graphite full-cells cycled in a cell voltage range 2.8 - 4.5 V (cathode mass loading: 12.2 mg cm^{-2}; rate: 1C (= 190 mA g^{-1}; N/P ratio = 1.35/1.00). Results correspond to the data shown in **Figure 3d-f**.

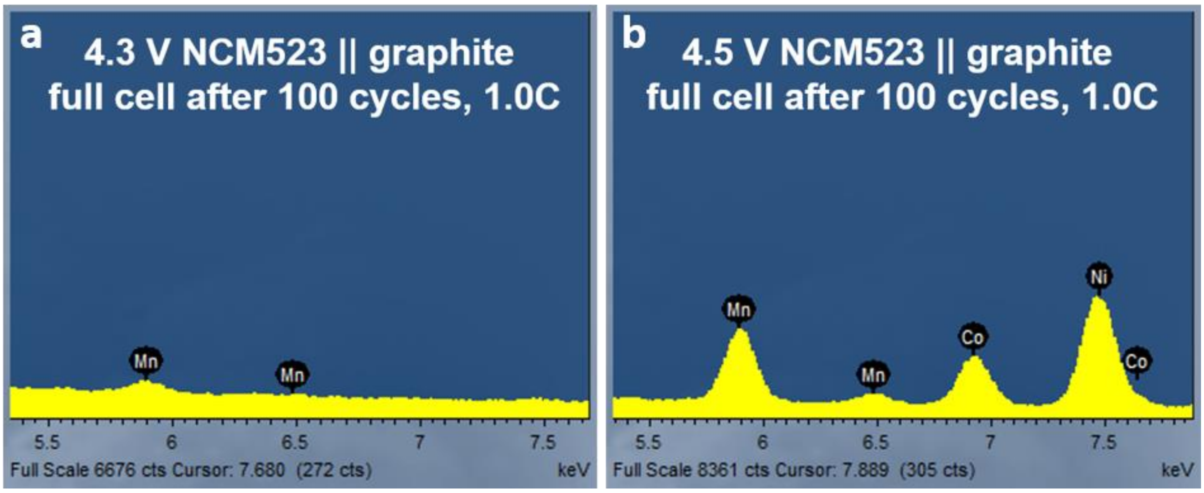

Figure S7. EDX elemental mappings of Ni, Co and Mn at the surface of cycled graphite anodes after 100 cycles, operated in NCM523 || graphite full-cells in cell voltage ranges of 2.8 - 4.3 V (a) and 2.8 - 4.5 V (b). Results correspond to the data shown in **Figure 2**.

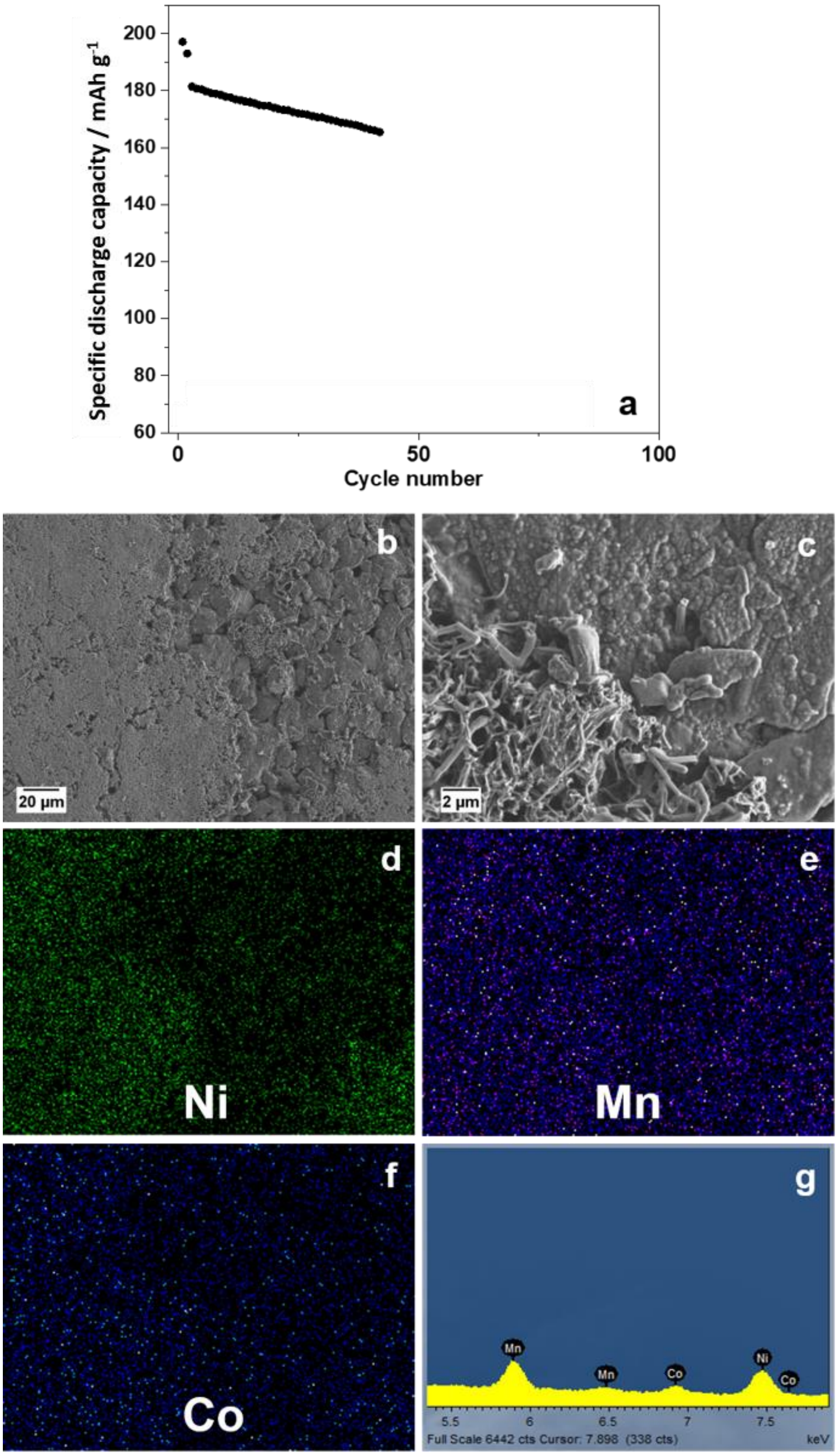

Figure S8. (a) Charge/discharge cycling performance of NCM523 || graphite full-cells (coin cells, two-electrode configuration) in a cell voltage ranges of 2.8 - 4.5 V (cathode mass loading: 12.2 mg cm^{-2}; charge/discharge cycling rate: 1C (= 190 mA g^{-1}); N/P ratio = 1.35/1.00); (b-g) SEM/EDX analysis of graphite negative electrodes after 40 cycles. Section (c) shows the enlarged area of the SEM image in (b), while sections (d-f) display the EDX elemental mappings of Ni (d), Mn (e) and Co (f) of the selected area of (c, g).

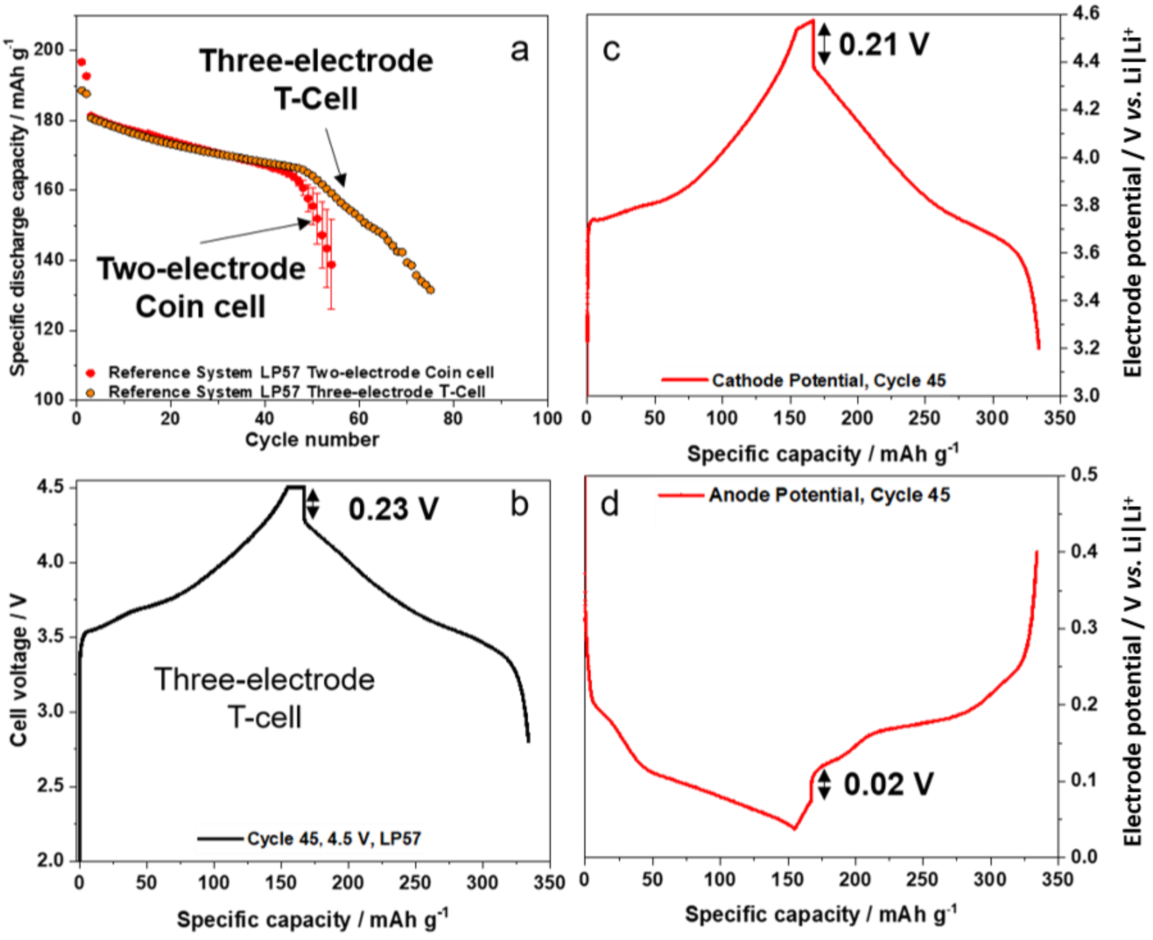

Figure S9. (a) Comparison of the charge/discharge cycling performance of NCM523 || graphite full-cells in coin cells (two-electrode configuration) and T-cells (three-electrode configuration) in a cell voltage range of 2.8 - 4.5 V (cathode mass loading: 12.2 mg cm^{-2}; charge/discharge cycling rate: 1C = 190 mA g^{-1}; N/P ratio = 1.35/1.00). (b) Charge/discharge cell voltage profile of the T-cell (45th cycle). (c) NCM523 cathode potential profile of the T-cell (45th cycle). (d) Graphite anode potential profile of the T-cell (45th cycle).

Three-electrode T-cells with Li metal as reference electrode (RE) were assembled to study the individual anode and cathode potentials. The cycling performance of NCM523 || graphite full-cells in two-electrode coin cells and three-electrode T-cells are depicted in **Figure S9a** and show a very similar cycling behavior, including the strong capacity drop (rollover failure) after ≈50 cycles. The voltage drop in the T-cell amounts ≈0.23 V (45th cycle), which is slightly lower than in two-electrode coin cells (0.32 V) but still very pronounced (**Figure S9b**). Further, the cathode potential shows a drop of ≈0.21 V (45th cycle), whereas the anode potential drop is only ≈0.02 V, indicating that the main part of cell impedance is caused by the cathode (**Figure S9c**, **d**). One possible explanation could be that large amounts of lithium are trapped in the SEI layer at the anode, caused by TM-induced SEI growth, which cannot migrate back to the cathode during discharge, causing further side reactions. It also has to be mentioned that the

high resistance growth of cathode materials during high-voltage operation is well-known.[3, 4] Despite the observed thick Li metal dendrites at 4.5 V, the average anode potential remains >0 V vs. Li|Li^+ (**d**). However, the measured anode potential is the overall anode potential, not considering local potentials. For example, the anode potential of the surface can be negative (≤0 V vs. Li|Li^+), while the other parts of the electrode (middle part of the electrode and electrode parts near current collector) will stay positive (≥0 V vs. Li|Li^+), so that the overall anode potential is positive, while there are local Li plating reactions.[5, 6] Therefore, it is unlikely to get unambiguous information about local dendritic Li metal deposits just on the basis of the overall measured anode potential. Similar observations were reported by Li et al.[7], showing that Li metal structures can be formed at graphite anodes in the presence of TM species via "underpotential deposition", i.e., referring to a reduction of metal ions at potentials more positive than the overall Nernst potential.

Further results of the chapter 2.2: "Impact of Charge-Discharge Rate Variation and Cathode Mass Loading Variation on Cycling Performance of NCM523 || Graphite Full-Cells"

The dendrite deposition morphology at 0.5C (4.5 V) differs from the observed dendrite "islands" at 1C (4.5 V, **Figure 3d-f**), which can be explained by a reduced charge/discharge rate and, thus a reduced amount of charge (Li^+ + e^-) available for intercalation as well as for decomposition product and Li metal dendrite formation within the same time frame. At 0.5C, only half of the charge is shuttled to the anode within the same time under constant current charging (i.e., the time for one CC-CV charge cycle is strongly increased, while the time for constant current charging is doubled), resulting in a less localized, broader distribution of Li metal dendrites, which results in more compact Li dendrite deposits (**Figure S10c**). However, the graphite particles surrounding the Li dendrites look very similar compared to the covered graphite particles observed at 1C (**Figure 3f** and **Figure S10d**). The species covering the graphite particles consist of many small spherically shaped deposits, which are agglomerated to larger structures.

The anode surface analysis was concentrated on two different spots, which were analyzed by SEM and EDX (**Figure S10b**). At spot 1, the graphite particles are highly covered by a thick decomposition layer and Li metal dendrites. Here, the EDX elemental mapping shows a high accumulation of the three TMs (Ni, Co and Mn), as seen in **Figure S10f** and **g** (due to the similar observations in regard to Co and Mn, only Ni is depicted), which is similar to the observed accumulation of the TMs at 1C (**Figure 3h-j**). Further EDX results are shown in **Figure S11**, illustrating high amounts of Ni, Co and Mn in the area of spot 1, while only small amounts of Mn are found at spot 2 (see (**Figure S10b)**. In contrast, at spot 2 (**Figure S10h** and **i**) no Li metal dendrites are found and the graphite particles are less covered by a decomposition layer, thus, only show an accumulation of Mn. However, this does not mean that Ni and Co are not present at the surface, it only means that their concentrations under the EDX detection limit.

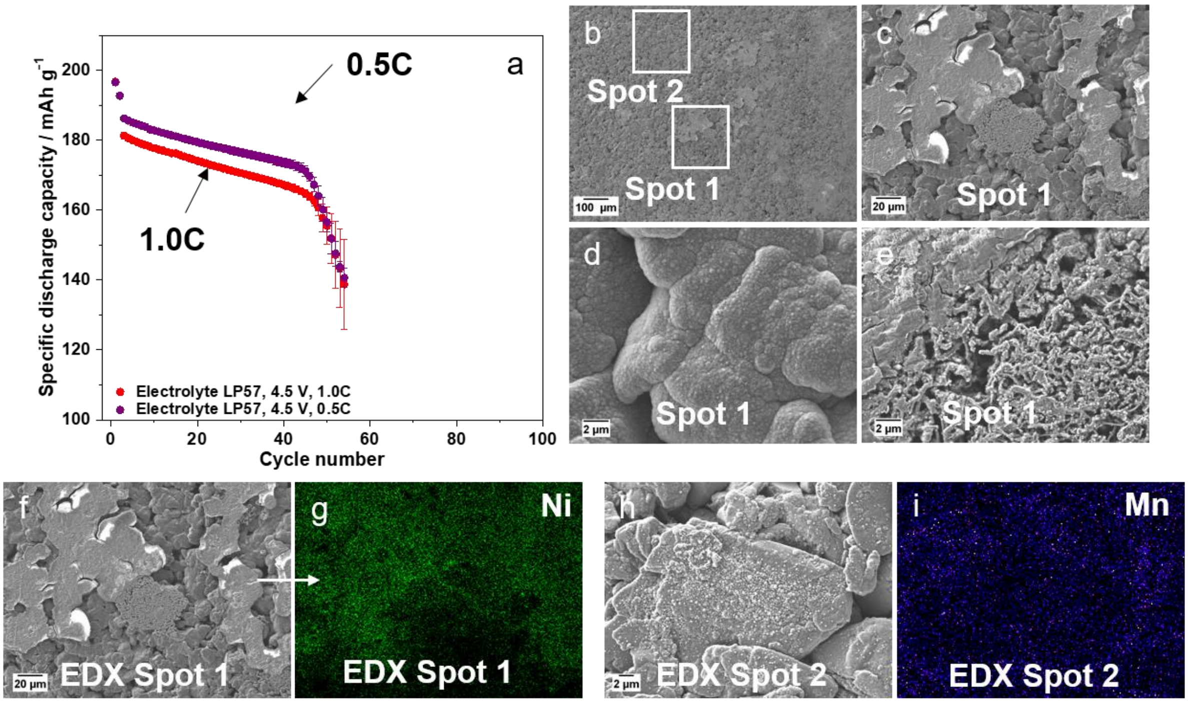

Figure S10. (a) Comparison of the charge/discharge cycling performance of NCM523 || graphite full-cells (coin cells, two-electrode configuration) in a cell voltage range of 2.8 - 4.5 V at charge/discharge rates of 1C and 0.5C (1C = 190 mA g^{-1}; cathode mass loading: 12.2 mg cm^{-2}; N/P ratio = 1.35/1.00) and SEM/EDX analysis of the graphite negative electrode after cycling at 0.5C (b-i). Sections (b-e) depict SEM images of the cycled graphite electrodes. Sections (f, g) show the enlarged area of the SEM image in (b; spot 1) and the corresponding EDX mapping of Ni (spot 1). Sections (h, i) display the enlarged area of the SEM image in (b; spot 2) and the corresponding EDX mapping of Mn (spot 2). Further EDX results are shown in **Figure S11**.

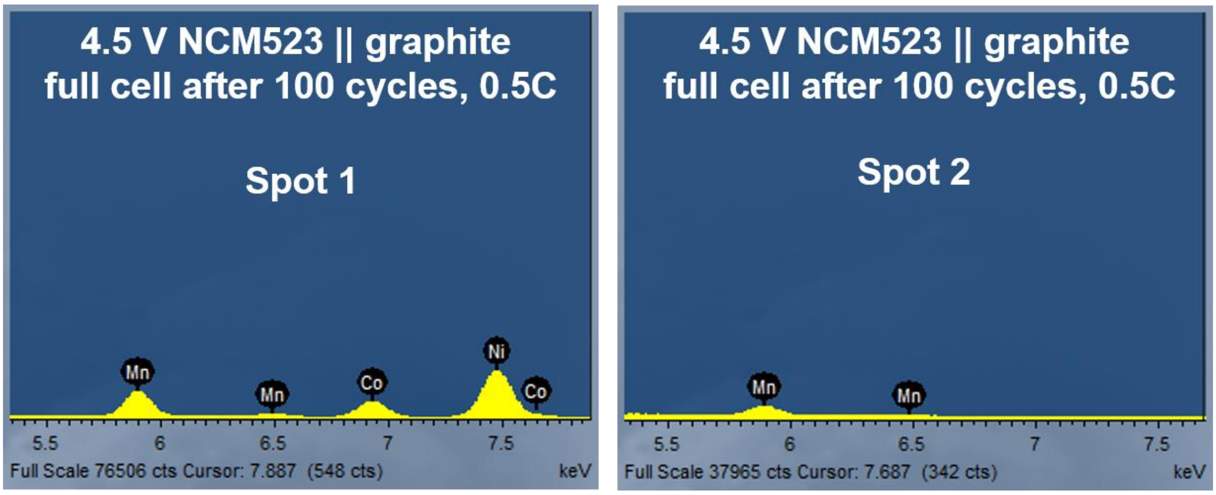

Figure S11. EDX elemental mappings of Ni, Co and Mn at the surface of cycled graphite anodes after 100 cycles, operated in NCM523 || graphite full-cells in a cell voltage range of 2.8 - 4.5 V (0.5C) at two different spots at the graphite anode surface (see **Figure 5b**). Results correspond to the data shown in **Figure 5**.

Another point which needs to be addressed, is the fact that a high C-rate (in combination with low operation temperature) can result in Li metal plating at graphite.[8] We showed that the amount of Li metal deposits is rather low at the aged graphite anode for NCM523||graphite full-cells cycled at 4.3 V and 1C, especially considering that we explicitly chose a position where we found some Li metal deposits (see **Figure 3b,c**), while other electrode spots showed no Li metal deposits (**Figure S3**). In contrast, at 4.5 V (0.5C or 1C) thick Li metal deposits were found all over the electrode (**Figure 3d-f** and **Figure S10**). In this respect, an even higher C-rate, such as 2C (at a cut-off of 4.5 V), can even prevent the early induced rollover failure (**Figure S12a**), while the discharge capacity drops to lower values. Nevertheless, very homogeneous Li metal deposits can be observed at the graphite anodes after 100 cycles (**Figure S12b, c**), which is another indication for the failure mechanism (I), as proposed in **Figure 8e**.

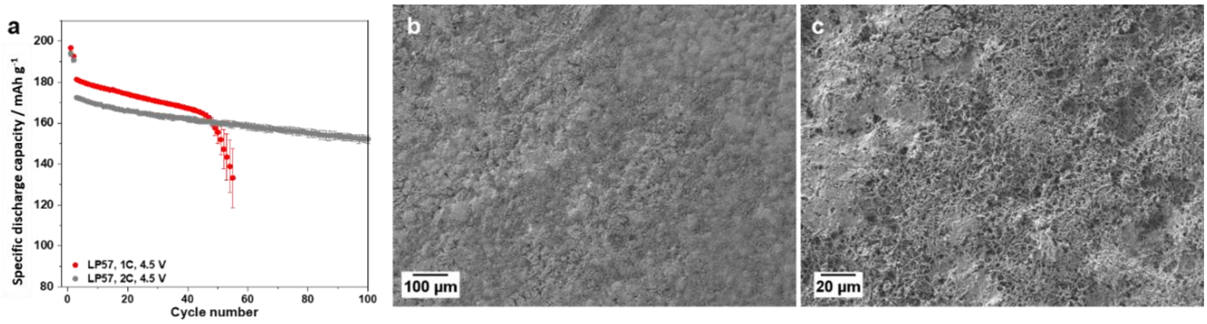

Figure S12. (a) Comparison of the charge/discharge cycling performance of NCM523 || graphite full-cells (coin cells, two-electrode configuration) in a cell voltage range of 2.8 - 4.5 V (cathode mass loading: 12.2 mg cm^{-2}; charge/discharge cycling rate: 1C and 2C (1C= 190 mA g^{-1} at 4.5 V); N/P ratio = 1.35/1.00); (b, c) SEM images of the homogenous Li metal deposition at the graphite anode after 100 charge/discharge cycles at 2C.

Further results of the chapter 2.3: "Impact of the Inhomogeneous Li metal Deposition on the Cell Performance"

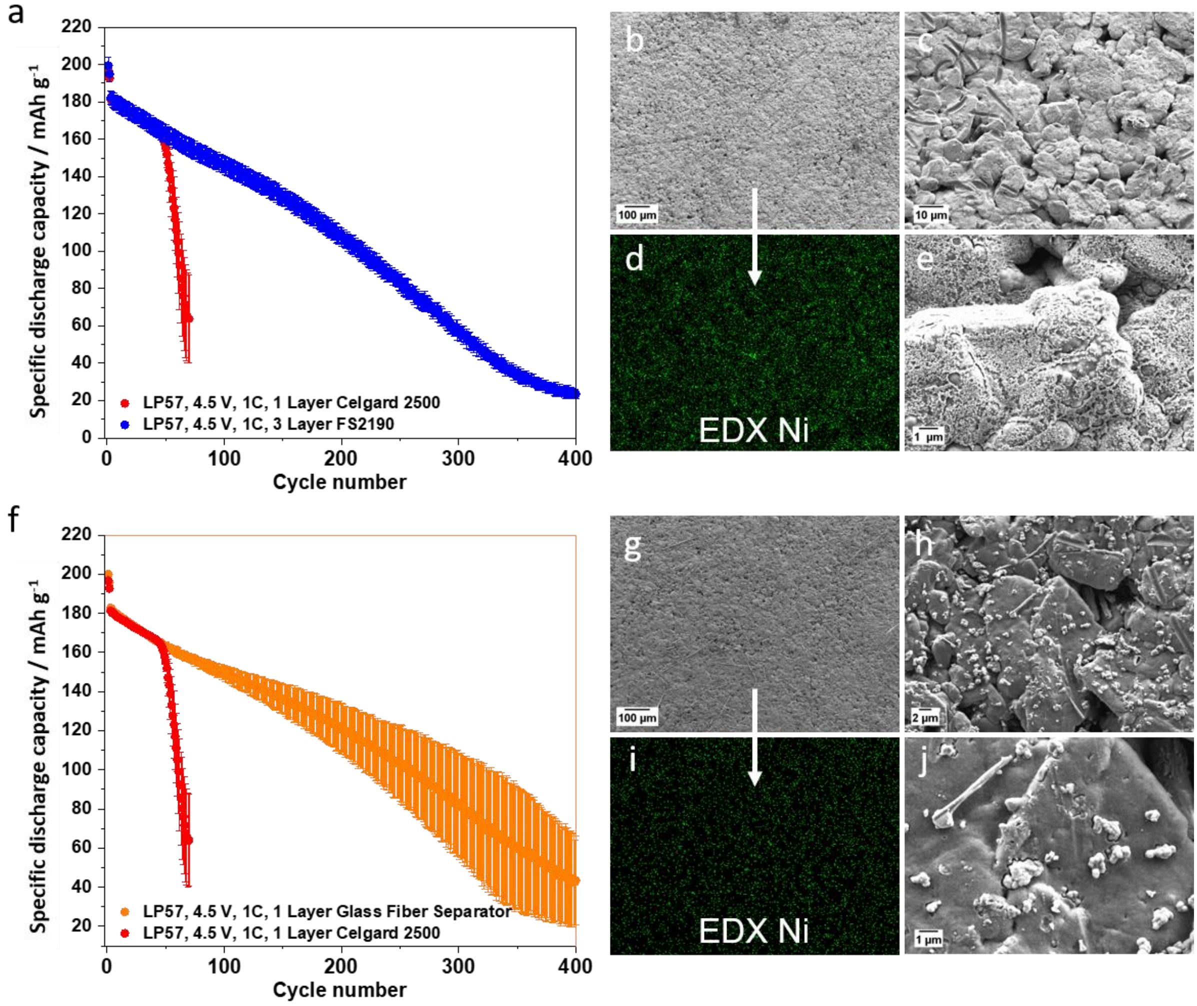

Figure S13. (a, f) Comparison of the charge/discharge cycling performance of NCM523 || graphite full-cells (coin cells, two-electrode configuration) in a cell voltage range of 2.8 - 4.5 V (cathode mass loading: 12.2 mg cm^{-2}; charge/discharge cycling rate: 1C (= 190 mA g^{-1} at 4.5 V); N/P ratio = 1.35/1.00) using different separators (i.e., 1 layer Celgard 2500, 1 layer glass fiber separator (Whatman) and 3 layers of a polyolefine separator (Freudenberg FS2190)); Sections (b-e) and (g-j) depict SEM/EDX images of the cycled graphite electrodes after 400 cycles using the polyolefine separator (b-e) and the glass fiber separator (g-j).

To further examine why the thicker separators suppress charge capacity fluctuations and voltage noise, the cycled graphite anodes from the NCM523 || graphite full-cells with the polypropylene (**Figure S13a-e**) and glass fiber (**Figure S13f-j**) separators were collected after 400 cycles and studied via SEM and EDX. The use of thick separators results in the formation of very homogenous Li metal deposits and uniform TM deposit distribution as well as very thick decomposition layers in both cases, however, no thick "dendrite islands", as previously

detected for Celgard-based cells, were observed. These observations can explain why no rollover failure was observed for these cells, as most likely no needle-like Li metal dendrites were formed, penetrating the separator, and resulting in short-circuits. Instead, severe capacity loss can be ascribed to the significant active lithium losses based on the parasitic SEI-forming reactions of the Li metal deposits and the electrolyte.

Further results of the chapter 2.4: "Mechanistic Elucidation of the High-Voltage Induced Cell Failure of NCM523 || Graphite Full Cells: Initial state of Li metal deposition"

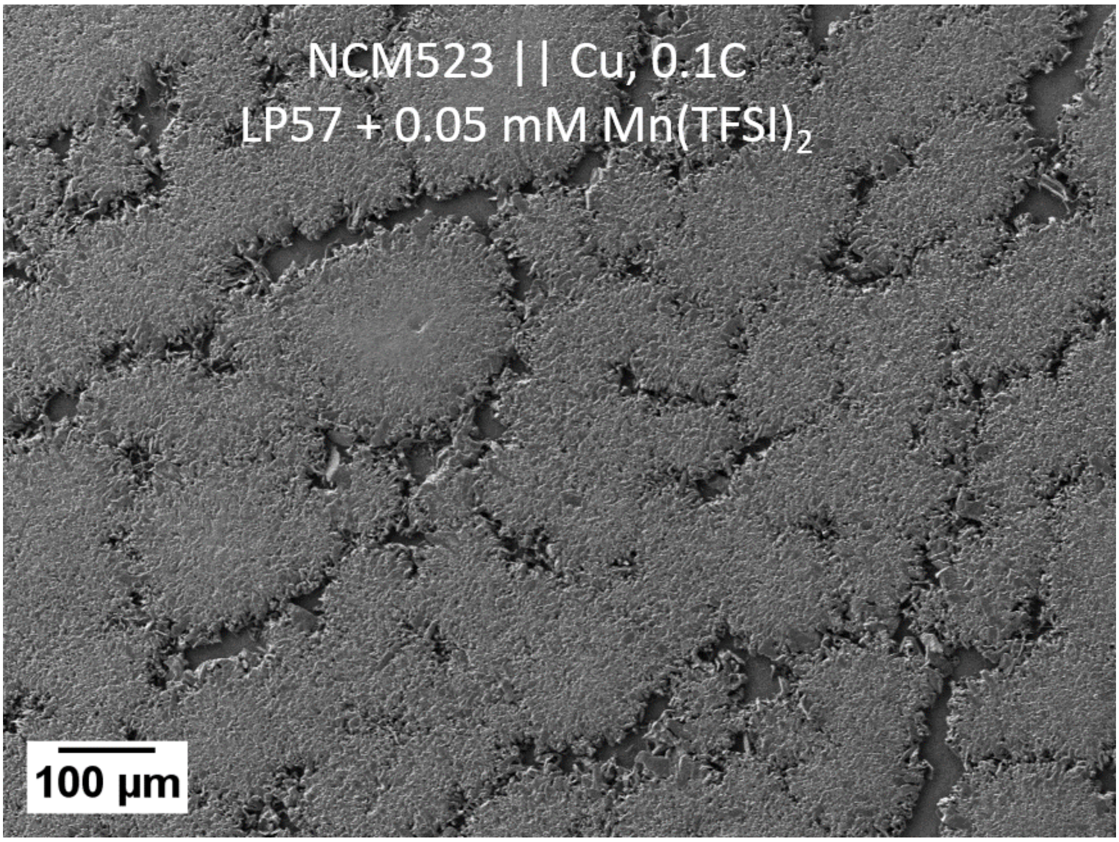

Figure S14. SEM image of copper foil obtained after the first charge to 4.2 V (at 0.1C) in NCM523 || Cu cells using LP57 + 0.1 mM $Mn(TFSI)_2$ electrolyte.

Further results of the chapter 2.5: "Mechanism for High-Voltage Induced Cell Failure of LIB Cells Caused by TM Induced Li Metal Growth at the Graphite Anode: The Worst-Case Scenario"

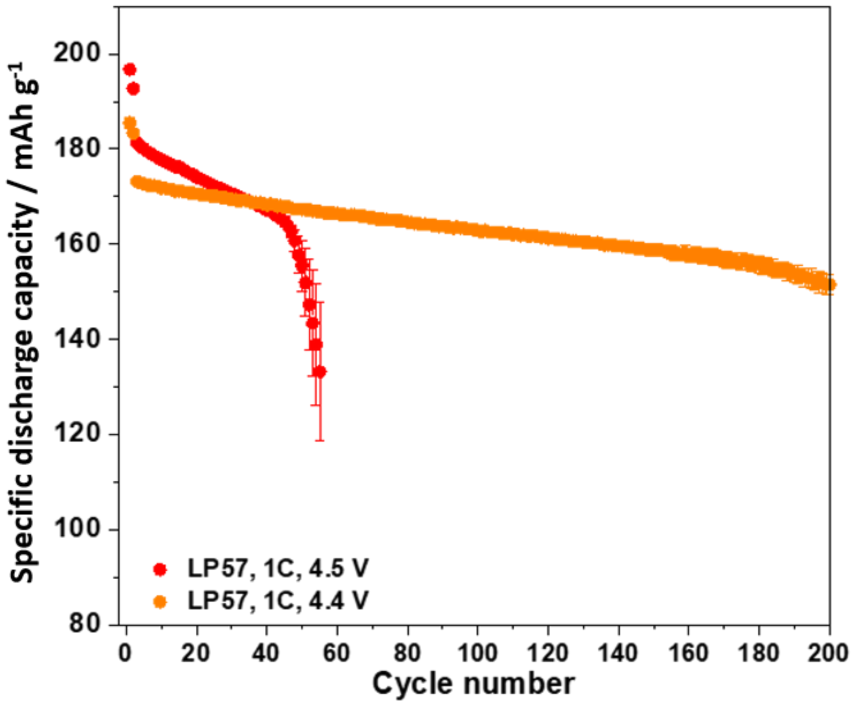

Figure S15. Comparison of the charge/discharge cycling performance of NCM523 || graphite full-cells (coin cells, two-electrode configuration) in cell voltage ranges of 2.8 - 4.4 V and 2.8 - 4.5 V (N/P = 1.35/1.00; cathode mass loading: 12.2 mg cm^{-2}; charge/discharge cycling rate: 1C (= 180 mA g^{-1} at 4.40 V; = 190 mA g^{-1} at 4.5 V).

Further results of the chapter 2.6: “Factors Influencing the Cycle Life of High-Voltage LIB Cells”

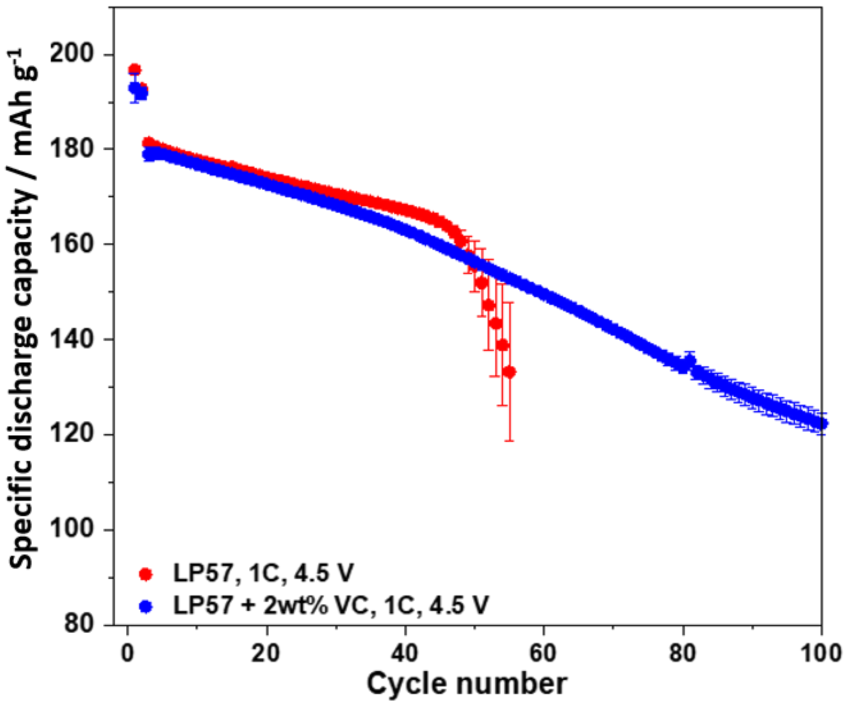

Figure S16. Comparison of the charge/discharge cycling performance of NCM523 | | graphite full-cells (coin cells, two-electrode configuration) in the cell voltage range of 2.8 - 4.5 V (N/P = 1.35/1.00; cathode mass loading: 12.2 mg cm^{-2}; charge/discharge cycling rate: 1C (= 190 mA g^{-1}). Two different electrolytes are used: LP57 and LP57 + 2 wt.% vinylene carbonate (VC).

References

1. B. Vortmann-Westhoven, M. Winter,S. Nowak, *J. Power Sources*, **2017**, *346*, 63-70.
2. M. Evertz, J. Kasnatscheew, M. Winter,S. Nowak, *Analytical and Bioanalytical Chemistry*, **2019**, *411*, 277–285.
3. J. Ahn, E. K. Jang, S. Yoon, S.-J. Lee, S.-J. Sung, D.-H. Kim,K. Y. Cho, *Appl. Surf. Sci.*, **2019**, *484*, 701-709.
4. D. W. Abarbanel, K. J. Nelson,J. R. Dahn, *J. Electrochem. Soc.*, **2016**, *163*, A522-A529.
5. X.-G. Yang, Y. Leng, G. Zhang, S. Ge,C.-Y. Wang, *J. Power Sources*, **2017**, *360*, 28-40.
6. M. Winter, K. C. Möller,J. O. Besenhard, *Lithium Batteries - Science and Technology (Eds.: Nazri, G.; Pistoia, G.)*, **2003**, 144-194.
7. W. Li, U.-H. Kim, A. Dolocan, Y.-K. Sun,A. Manthiram, *ACS Nano*, **2017**, *11*, 5853-5863.
8. Q. Q. Liu, R. Petibon, C. Y. Du,J. R. Dahn, *J. Electrochem. Soc.*, **2017**, *164*, A1173-A1183.
